# The Effect of Aza-Glycine Substitution on the Internalization of Dabcyl-Containing Short Oligoarginine

**DOI:** 10.3390/biomedicines14051025

**Published:** 2026-04-30

**Authors:** Karima Tarchoun, Dóra Soltész, Ildikó Szabó, Jong-Won Song, Ho-Jin Lee, Zoltán Bánóczi

**Affiliations:** 1 Department of Organic Chemistry, Institute of Chemistry, Faculty of Science, ELTE Eötvös Loránd University, Pázmány Péter Sétány 1/A, 1117 Budapest, Hungary; tarchoun.karima14@gmail.com (K.T.); soltesz.dora6@gmail.com (D.S.); 2 Hevesy György PhD School of Chemistry, Institute of Chemistry, ELTE Eötvös Loránd Uniersity, Pázmány Péter Sétány 1/A, 1117 Budapest, Hungary; 3 HUN-REN-ELTE Research Group of Peptide Chemistry, 1117 Budapest, Hungary; ildiko.szabo@ttk.elte.hu; 4 Department of Chemistry Education, Daegu University, Daegudae-ro 201, Gyeongsan-si 38453, Gyeongsangbuk-do, Republic of Korea; sjoshua@daegu.ac.kr; 5 Division of Natural and Mathematical Sciences, LeMoyne-Owen College, Memphis, TN 38126, USA; ho-jin_lee@loc.edu

**Keywords:** cell-penetrating peptide, CPP, oligoarginine, aza-amino acid, aza-peptide, flow cytometry, cellular uptake

## Abstract

**Background/Objectives**: Longer oligoarginines are very effective cell-penetrating peptides. It has been shown that a minimal number of positively charged side chains is necessary for efficient cellular uptake. But a highly positively charged peptide may interact with its cargo molecule, thereby reducing its efficiency. Several chemical modifications were tested to improve the internalization of short tetraarginine derivatives. Aromatic groups, such as Dabcyl at the N-terminus, Trp in the sequence, and AMBA or PABA in the backbone, were used to improve internalization. The other useful modification was the aza-glycine substitution in the case of penetratin. **Methods**: In this study, the effect of aza-glycine insertion into the peptide Dabcyl-RRRRK(Cf) on internalization was studied and compared with that of the Trp-modified peptide Dabcyl-RRWRRK(Cf). To explain the noticed difference in the biological activity of peptides, DFT calculations and the prediction of membrane-binding free energy (ΔΔF) from a peptide sequence were performed. **Results**: It turned out that the position of the aza-glycine moiety does not have an influence on the cellular uptake. The aza-glycine-containing peptide showed higher internalization than the Dabcyl-RRRRK(Cf) peptide. Besides this, these peptides have similar or higher cellular uptake than that of octaarginine at lower concentrations (c < 2 µM). The aza-glycine affected not only cellular uptake but also the entry mechanism. The structure of peptides depended on the amino acids (Trp, Gly, or azaGly) in their sequences and their positions. **Conclusions**: These may result in the different amphiphilicity of peptides, and thus changes in the hydrophobic moment and in the binding affinity of peptides to the negatively charged membrane surface.

## 1. Introduction

The cell membrane acts as a dynamic barrier, selectively controlling the transport of compounds necessary for the cell to maintain homeostasis, and it provides protection for the interior of cells. Specific molecules, such as small hydrophobic molecules and gases like oxygen and carbon dioxide, can diffuse across the membrane, whereas others, such as highly hydrophilic or charged molecules (sugars, amino acids, etc.) cannot. This barrier function of the cell membrane affects the intracellular delivery of biologically active molecules and makes their application more challenging. In many cases, the reduced cellular uptake jeopardizes the in vivo biological activity. To avoid these drawbacks, chemical modifications or alternative drug delivery systems can be used. They may result in efficient transport and overcome the cell membrane barrier-caused inhibition of drug uptake. Sometimes, just a little chemical modification (replacement of an amide with a thioamide in a macrocyclic peptide) can enhance the permeability and bioavailability [1]. A particular type of drug delivery system is the group of cell-penetrating peptides (CPPs). These short peptides (less than 30 amino acids) have been the focus of lot of research in recent years [2]. This is due to their ability to translocate across the cell membrane and carry several kinds of molecules, e.g., peptides [3,4], proteins [5], small drug molecules [6], and oligonucleotides [7]. The firstly discovered and most well-known examples of CPPs are Tat (GRKKRRQRRRPPQ), derived from the human immunodeficiency virus (HIV-1) [8,9], and penetratin (RQIKIWFQNRRMKWKK), which was found in the *Drosophila* Antennapedia homeodomain [10]. These peptides have been extensively studied and have undoubtedly demonstrated their value as delivery vectors. It was revealed that the Tat peptide, termed as TAT48-60, part of the TAT protein, is the key factor responsible for the efficient cellular uptake of the protein [9,11], and a similar role was shown for penetratin. It was found later that their high cellular uptake is due to the high abundance of Arg residues [12] and their positively charged guanidinium group [13]. Among the various oligomers of positively charged amino acids (oligolysines, oligoarginines, and oligohistidines), oligoarginines (octaarginine (Arg_8_)) had the highest cellular uptake, proving the importance of Arg residues [14]. This was explained by the specific interaction between guanidino and phosphate groups in the membrane lipids [15]. Octaarginine as a carrier for a wide range of cargos and peptides was thoroughly examined by our research group [16]. Although it was successfully applied in many cases, it had poor ability to deliver methotrexate and its pentaglutamylated derivative [17]. It is well known that shorter oligoarginines have weaker or no cellular uptake. Enhancing the hydrophobicity of a peptide can improve its internalization; norbornene-based polymers, which exhibit CPP-like properties, demonstrated a three-fold increase in uptake when alkyl groups were added to the side chains [18]. Longer unsaturated fatty acids could enhance the internalization of tetraarginine, which is an inefficient CPP [19]. The modification of the N-terminus of tetraarginine by hydrophobic aromatic amino acids (L-phenylalanine or/and L-2-naphthylalanine) could improve its cellular uptake [20]. In the case of cyclic tetraarginine, modifications with both aromatic and alkyl groups could enhance the internalization; the decanoyl group showed the most significant effect [21]. The aromatic hydrophobic amino acid, tryptophan (Trp) is known for its positive effect on the cellular uptake of CPPs. Its impact has been widely studied, particularly focusing on its position and its number within a sequence [22]. For instance, the introduction of Trp into an oligoarginine (Arg8-12) improves its internalization, and its effect is greater when it is placed into the middle of a sequence rich in Arg and Trp residues (RRWWWWRR) [23]. The Dabcyl group (4-((4-(dimethylamino)phenyl)azo)benzoyl) is a well-known dark quencher commonly employed in FRET systems [24]. Micelles containing Dabcyl-labeled RNA-polymer amphiphiles were found to internalize more effectively than micelles without the Dabcyl group [25]. The introduction of this hydrophobic moiety at the N-terminus of tetra- and hexaarginine demonstrated a significant increase in the cellular uptake [16], and a similar effect was reported in the case of decaarginine [26]. We showed earlier that the modification of Dabcyl-containing tetraarginine by incorporating one or two Trp residues at different positions increased the internalization of all peptides; the most promising ones are Dabcyl-WRRRRK(Cf) and Dabcyl-RRWRRK (Cf) [27].

**Figure 1:**
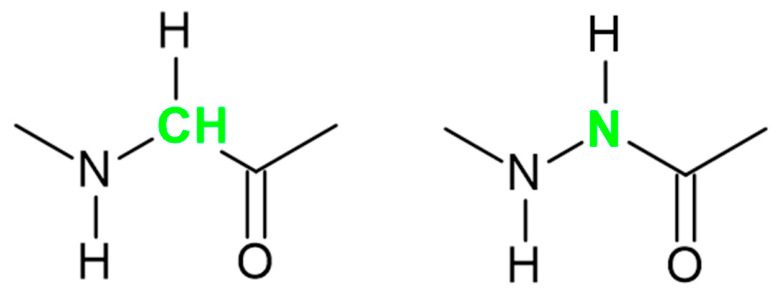
Chemical structure of glycine and aza-glycine counterpart.

In addition, the changed hydrophilicity/hydrophobicity structural modifications may also enhance the internalization of CPPs. They can be chemical modifications of the peptide backbone to develop peptide mimics [22,28]; substituting one or more amino acids with an aza-amino acid is a promising strategy. In these peptide mimics the C^α^ atom of the amino acid is replaced by nitrogen, resulting in an aza-peptide (Figure 1) [29].

Our recent work showed that the aza-glycine substitution of Trp near the C-terminus in penetratin led to a promising delivery vector, Cf-RQIKIWFQNRRK-azaGly-KK, with higher cellular uptake [30]. Spectroscopic [31,32], computational [31,33,34], and crystallographic [34,35,36] studies have demonstrated that aza-peptides can induce turn conformations in the structure of the parent peptides. Moreover, this substitution may also improve the biological activity, pharmacokinetic, and pharmacological properties of the original peptide. For instance, the substitution of glycine by its counterpart aza-glycine in the Arg-Gly-Asp (RGD) peptide motif, which plays a key role in cell adhesion, showed that it may affect both activity and selectivity [37]. However, the impact of smaller amino acid analogs on CPP uptake and stability remains poorly understood. Aza-glycine is a simple and easy-to-prepare moiety, which can be readily introduced into peptides. Both its coupling to a normal amino acid and the coupling of a normal amino acid to aza-glycine are generally less challenging than with other aza-amino acid residues. Aza-glycine, with its reduced steric bulk compared to other aza-amino acids, may enhance internalization while maintaining peptide stability. In this paper, we described the synthesis and characterization of various modified Dabcyl-containing tetraarginine peptides. We were interested in whether the substitution of the Trp residue by aza-glycine or two aza-glycine residues may enhance the internalization of the poorly penetrating CPP Dabcyl-tetraarginine. We assumed that the combination of the unnatural Dabcyl residue and azaGly could improve the cellular uptake of short oligoarginines. In the first set of peptides, the Trp residue was replaced by one azaGly in the middle, near the C-termini or near the N-termini, to study the influence of this substitution on the internalization of peptides *Dabcyl*-RRWRRK(*Cf*), *Dabcyl*-RRRRWK(*Cf*), and *Dabcyl*-WRRRRK(*Cf*). The effect of aza-glycine substitution of Trp in peptides *Dabcyl*-RRWWRRK(*Cf*), *Dabcyl*-RRWRRWK(*Cf*), and *Dabcyl*-WRRWRRK(*Cf*) was studied too. Fluorescent labeling was carried out on the ε-amino group of a lysine residue, which was added to the C-terminus using 5(6)-carboxyfluorescein (Cf). The cellular uptake was studied on the A-431 cell line using flow cytometry. The peptide *Dabcyl*-RRRRK(*Cf*) was used as a reference. The concentration dependence and the mechanism of entry of selected constructs were also examined. To examine structural changes behind the effect of aza-glycine substitution, DFT calculations were performed.

## 2. Materials and Methods

### 2.1. Synthesis of Peptides

Peptide synthesis was performed manually using solid-phase peptide synthesis (SPPS) on Rink amide MBHA resin (0.1 g, 0.65 mmol/g) following the Fmoc/*t*Bu strategy. The amino acids were introduced as Fmoc-Arg(Pbf)-OH, and Fmoc-Lys(Boc)-OH (where Fmoc is fluorenylmethyloxycarbonyl; Pbf is 2,2,4,6,7-pentamethyldihydrobenzofuran-5-sulfonyl; and Boc is the tert-butyloxycarbonyl). The temporary Fmoc-protecting group was removed using a cleavage solution containing 2% piperidine and 2% 1,8-diazabicyclo [5.4.0]undec-7-ene (DBU) in N,N-dimethylformamide (DMF) (2 + 2 + 5 + 10 min). Then, the resin was washed with DMF (8 × 1 min). Coupling reactions were carried out in DMF for 60 min at room temperature. The amino acids, N,N-diisopropylcarbodiimide (DIC), and ethyl(hydroxyimino)cyanoacetate (OxymaPure) as coupling reagents were used in 3 equivalent excesses. The aza-glycine residue was introduced into the peptide chain by the activation of the Fmoc-hydrazide moiety in DMF solution with carbonyldiimidazole (CDI), and in the presence of 2 eqv of N,N-diisopropylethylamine (DIEA), forming an aza-glycine donor [38]. To ensure the success of the coupling of each amino acid, a ninhydrin test was conducted except for the coupling to the aza-glycine moiety. Therefore, that amino acid derivative was coupled twice. After the last Fmoc group was removed, the Dabcyl group was attached to the N-terminus using the same coupling reagents (DIC/Oxyma pure), followed by the Kaiser test. The peptide resin was washed with DMF and dried with DCM (3 × 3 min). The peptides were cleaved from the resin using a mixture containing 5 mL TFA, 0.365 g phenol, 0.25 mL distilled water, 0.25 mL thioanisole, and 0.125 mL 1,2-ethanedithiol as scavengers. The crude products were precipitated with dry diethyl ether, then dissolved in 10% acetic acid, followed by lyophilization for a night. The crude peptides were purified by semi-preparative RP-HPLC. The purity of peptides was verified by analytical RP-HPLC, and they were identified by ESI-MS. Cf was attached to the ε-amino group of a lysine residue in the solution phase. These reactions were carried out in DMF using 1 eqv of DIC and Oxyma pure as coupling reagents in the presence of 2 eqv of DIEA for overnight. The labeled peptides shown in Table 1 were purified and identified as the peptides were (analytical RP-HPLC, semi-preparative RP-HPLC, and ESI-MS (Supplementary Materials, Figures S1–S26)).

### 2.2. Cellular Uptake of Peptides

For the cellular uptake studies, a total of 10^5^ A-431 cells (human skin squamous cancer cells), generously provided by Prof. József Tóvári (Institute of Oncology, Budapest, Hungary), per well were plated on 24-well plates. The cells were maintained at 37 °C for 24 h, followed by the treatment with peptide solutions at a 5 µM concentration in a serum-free medium for 90 min. As a negative control, cells were treated with serum-free medium for 90 min. After the incubation, the cells were washed and treated with 100 µL trypsin for 10 min to remove membrane-bound peptides and detach the cells from the plate. The addition of 900 µL HPMI buffer (glucose, NaHCO_3_, NaCl, HEPES, KCl, MgCl_2_, CaCl_2_, Na_2_HPO_4_ × 2 H_2_O) containing 10% fetal calf serum was used to terminate the activity of trypsin. Cells were subsequently transferred from the plates into FACS tubes, which were centrifuged at 216× *g* at 4 °C for 5 min. After resuspension in 250 μL HPMI, their fluorescence intensity was quantified using flow cytometry (LSR II, BD Bioscience, San Jose, CA, USA). The data analysis was conducted with FACSDiVa 5.0 software. For some peptides, the cellular uptake was determined at different concentrations (0.5 µM, 1 µM, 2 µM, and 5 µM) as well. The internalization pathway was studied using the same protocol that was applied in cellular uptake experiments, with the only difference being that the cells were pretreated with a solution of different inhibitors for 30 min. Macropinocytosis was inhibited using 5-(N-ethyl-N-isopropyl)amiloride (EIPA) [39], clathrin-mediated endocytosis was blocked with chlorpromazine (CPZ) [40], colchicine (Col) was used to evaluate the role of microtubules [41], and methyl-β-cyclodextrin (CyD) was employed to inhibit caveolae/lipid raft-mediated endocytosis [42]. To inhibit all endocytosis pathways, sodium azide (NaN_3_) (500 µM) and 2-deoxyglucose (DOG) (250 mM) were used. Three independent experiments were performed. The measured mean fluorescence intensity was normalized to the mean fluorescence intensity of control peptides at a given concentration.

### 2.3. Calculations

The density function theory (DFT) functional was used to calculate the conformational behavior of Trp, Gly, or azaGly-containing peptides: *Ac*-WRRRRK-*NHMe* (**1**), *Ac*-GRRRRK-*NHMe* (**2**), *Ac*--**azaGly-**RRRRK-*NHMe* (**3**), *Ac*-RRWRRK-*NHMe* (**4**), *Ac*-RRGRRK-*NHMe* (**5**), *Ac*-RR-**azaGly-**RRK-*NHMe* (**6**), *Ac*-RRRRWK-*NHMe* (**7**), *Ac*-RRRRGK-*NHMe* (**8**), and *Ac*-RRRR-**azaGly-**K-*NHMe* (**9**). Here, *Ac* represents the acetyl group at the N-terminus; *NHMe* represents the N-methyl amide group at the C-terminus; and the bold letter **azaGly** represents an aza-glycine residue. To obtain the initial structures of the model peptides, the Pep-Fold4 server [43] (accessed on 13 December 2024) was used at pH 7.5 and 0.1 M ionic strength. We chose the best structure among the five best structures of the model peptide. From the lowest energy conformer, the N-terminal and C-terminal functional groups were added using GaussView 6.1.1. The resulting peptides **1–9** were fully optimized at the SMD/LC-ωPBE-D3/3-21+G(d) level of theory using the Gaussian 16 program [44]. LC-ωPBE-D3 represents the long-range corrected hybrid functional combined with Grimme’s D3 empirical dispersion correction [45,46]. The SMD solvation model [47] was employed to account for the water effect. Due to the size of the model peptides, we used a small basis set, 3-21+G(d). The backbone (φ, ψ) dihedral angles of model peptides were examined to investigate the conformational changes (Supplementary Materials Figure S28). The figures were generated using GaussView 6 [48] and PyMol [49].

The PMIpred (Protein–Membrane Interaction predictor) server (https://pmipred.fkt.physik.tu-dortmund.de/) was used to predict the membrane interactions for the target peptides [50]. The relative membrane-binding free energy (ΔΔF) from a peptide sequence was obtained using a neural network model trained on molecular dynamics simulation data (as accessed on 8 January 2026) [51]. The peptides used for the prediction of sensing free energy in this work were the following: AGRRRRKA, ARRWRRKA, ARRGRRKA, ARRRRWKA, ARRRRGKA, ARRRRKA, ARRWWRRKA, ARRGGRRKA, AWRRWRRKA, AGRRGRRKA, ARRWRRWKA, ARRGRRGKA, and ARRRRRRRRA. The Ala residues at the N- and the C-termini were introduced to mimic the block imposed by the fluorescein functional group. The PMIpred server can predict ΔΔF and ΔF_sm (R = 50) (Table 2), where ΔΔF is the predicted curvature-sensing free energy, and ΔΔF_sm (R = 50) is the predicted membrane-binding free energy for a vesicle with a 50 nm radius [50,51,52,53,54]. The detailed definition of sensing and binding free energy from a peptide sequence can be found in the original work [51]. The hydrophobicity, hydrophobic moment, and binding free energy with negatively charged membranes (POPC/POPG) and neutral membranes (POPC) were calculated (SI Figure S28) [53,54]. In this server, a non-standard amino acid residue, aza-glycine, was not considered; we could not obtain the sensing free energy of the membrane.

### 2.4. Statistical Analysis

The results of the cellular uptake studies are presented as mean values ± standard deviation. Statistical analysis was conducted using Student’s *t*-test, with *p* values below 0.05 considered statistically significant.

## 3. Results

### 3.1. Synthesis of Labeled Peptides

Peptides rich in arginine (Arg), such as Tat, penetratin, and oligoarginines, are highly effective cell-penetrating peptides. The internalization of these peptides revealed a strong dependence on the number of Arg residues; more Arg residues led to greater cellular uptake. For penetratin, it was found that tryptophan (Trp) also plays a crucial role in its internalization process [10,55]. Our previous findings indicated that the substitution of tryptophan residue at position 48 of the penetratin with an azaGly residue (Trp48azaGlyPen(desMet)) resulted in significantly enhanced uptake and a different internalization pathway compared to the original peptide [30]. Several studies examined the impact of Trp on the cellular uptake of oligoarginines (octa- and hexaarginine), which possess a good to moderate cell-penetrating ability. It has been demonstrated that the Dabcyl group could increase the cellular uptake of hexa- and tetraarginine [16], and this effect could be further enhanced with the introduction of tryptophan [27]. The results showed that Trp in the middle of the sequence (*Dabcyl*-RRWRRK(*Cf*)) or at the N-termini (*Dabcyl*-WRRRRK(*Cf*)) could enhance the internalization. Based on our earlier findings, azaGly substitution of Trp may not only retain but also increase the cellular uptake of penetratin. Thus, we decided to study the same modification on Dabcyl and Trp-containing tetraarginine derivatives. In these peptides, the position and the number of azaGly varied. Peptides were manually synthesized on Rink-amide MBHA resin through solid-phase peptide synthesis. The azaGly residue was introduced using three equivalents of Fmoc-NH-NH_2_ and three equivalents of CDI in the presence of DIEA as a base [38]. Peptides were labeled by Cf in the solution phase on the ε-amino group of a C-terminal Lys residue using DIC and Oxyma pure in DMF. Labeled peptides were purified by preparative RP-HPLC. Analytical RP-HPLC and ESI-MS were used for the characterization (Table 1; the analytical RP-HPLC chromatograms and MS spectra are in the Supplementary Materials, Figures S1–S26).

### 3.2. Cellular Uptake

The cellular uptake of fluorescently labeled peptides (Table 1) was measured on A-431 human skin squamous cancer cells through flow cytometry. This cell line was used earlier to examine the cellular uptake of penetratin derivatives [30]. Cells were treated with a solution of peptides at 5 μM concentration for 90 min at 37 °C. This concentration was selected based on our earlier results, which indicated efficient cellular uptake without detectable cytotoxicity. The values of the fluorescence intensity were adjusted based on the autofluorescence intensity measured in untreated cells. The live/dead cell ratio was also determined through flow cytometry analysis, and it showed that none of the examined peptides had cytotoxicity, even at the highest concentration used (Supplementary Materials Figure S27).

**Figure 2:**
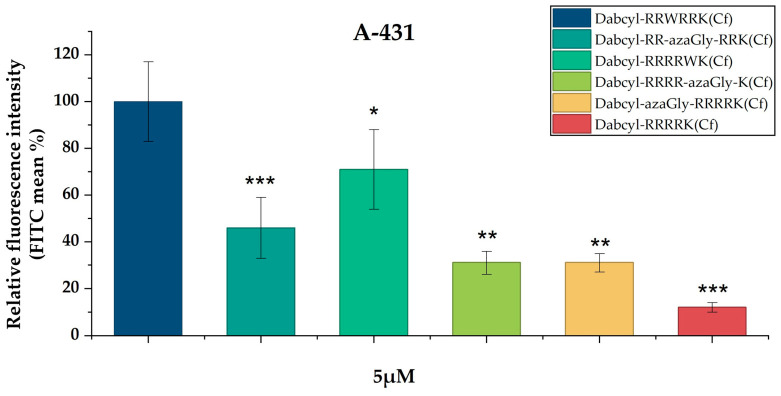
Comparison of the cellular uptake of Dabcyl-containing tetraarginine derivatives into A-431 cells. Cells were incubated with 5 µM peptide solution for 90 min at 37 °C. The fluorescence intensity of the cells was determined through flow cytometry. The fluorescence intensity is relative to *Dabcyl*-RRWRRK(*Cf*) at 5 µM (100%). Data represent the mean ± standard deviation (SD). Any significant difference in peptide internalization from the control was measured using Student’s *t*-test. The asterisks show a significant difference between the control peptide (Dabcyl-RRWRRK(Cf)) and its derivatives (* *p* < 0.05, ** *p* < 0.01, *** *p* < 0.001).

First, the effect of one azaGly substitution in different positions (middle, C-terminal, N-terminal) was examined. The cellular uptakes of these peptides were normalized to the fluorescence intensity of *Dabcyl*-RRWRRK(*Cf*) at 5 µM (100%) (the measured FITC values are presented in Figure S31). These azaGly-substituted peptides had lower cellular uptake than *Dabcyl*-RRWRRK(*Cf*). Their internalization was between 31 and 46% of the reference. Among them, *Dabcyl*-RR-azaGly-RRK(*Cf*) showed the highest internalization (46%). The internalization of the peptide *Dabcyl*-RRRRWK(*Cf*) with a Trp at the C-terminus was significantly lower (71% of the reference), indicating the crucial role of Trp position (Figure 2).

All of the azaGly-containing peptides showed a higher cellular uptake than the peptide *Dabcyl*-RRRRK(*Cf*) (Figure 2). Based on the data, the position of azaGly does not have an influence on the cellular uptake; all substituted peptides showed very similar fluorescence intensities.

**Figure 3:**
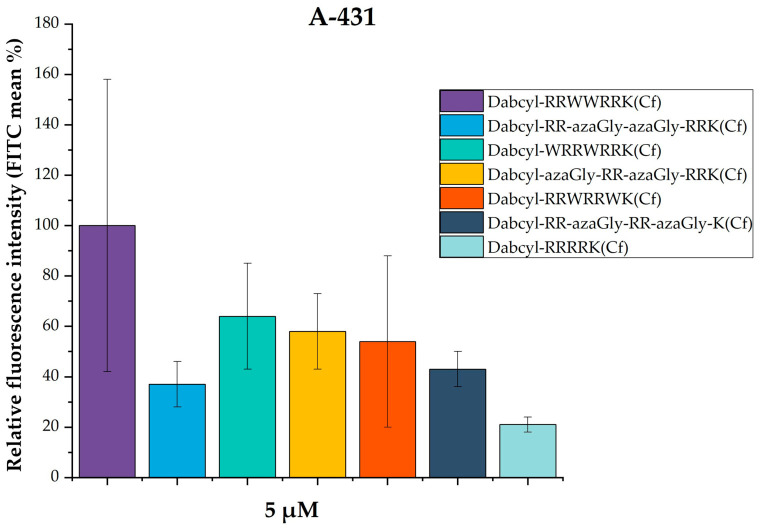
Study of the effect of azaGly substitution of Trp in different positions into Dabcyl-containing tetraarginine derivatives on the cellular uptake. A-431 cells were incubated with 5 µM peptide solution for 90 min at 37 °C. The fluorescence intensity of the cells was determined through flow cytometry. The fluorescence intensity is relative to *Dabcyl*-RRWWRRK(*Cf*) at 5 µM (100%). Data represent the mean ± standard deviation (SD). No significant difference in the peptide’s internalization from the control peptide (*Dabcyl*-RRWWRRK(*Cf*)) was detected using Student’s *t*-test.

In the next round, the effect of two azaGly residues at different positions was investigated (*Dabcyl*-RR-azaGly-azaGly-RRK(*Cf*), *Dabcyl*-RR-azaGly-RR-azaGlyK(*Cf*), *Dabcyl*-azaGly-RR-azaGly-RRK(*Cf*). The cellular uptakes of these peptides were normalized to the fluorescence intensity of *Dabcyl*-RRWWRRK(*Cf*) at 5 µM (100%) (the measured FITC values are presented in Figure S32). The results showed that all azaGly-containing peptides have lower internalization than the *Dabcyl*-RRWWRRK(*Cf*) (Figure 3). Their internalization ranged from 37% to 58% of this peptide. Among them, *Dabcyl*-azaGly-RR-azaGly-RRK(*Cf*) showed the highest uptake (58%), indicating a potential effect of the azaGly position and its distance from the Dabcyl group on cellular internalization. The two peptides with tryptophan residues (*Dabcyl*-WRRWRRK(*Cf*) and Dabcyl-RRWRRWK(Cf)) showed reduced internalization compared to the reference peptide *Dabcyl*-RRWWRRK(*Cf*), by 64% and 54%, respectively. In particular, the cellular uptake of *Dabcyl*-WRRWRRK(*Cf*) and its counterpart with aza-glycine, *Dabcyl*-azaGly-RR-azaGly-RRK(*Cf*), were comparable. The unmodified control peptide *Dabcyl*-RRRRK(*Cf*) demonstrated minimal cellular uptake (21%), confirming the limited internalization efficiency of tetraarginine in the absence of aromatic residues.

**Figure 4:**
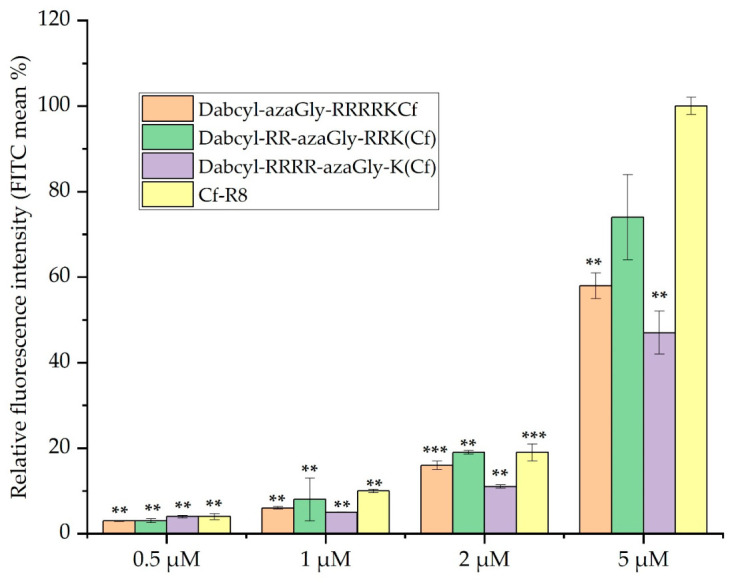
Concentration dependence of cellular uptake on A-431 cells. The cells were treated with peptides at 0.5, 1, 2, and 5 μM concentrations at 37 °C for 90 min. Then, they were trypsinized, and the fluorescence of the cells was investigated through flow cytometry. The fluorescence intensity is relative to Cf-R_8_ at 5 μM (100%). The difference between the labeled peptides and Cf-R_8_ was determined by Student’s *t*-test (** *p* < 0.01, *** *p* < 0.001). Data represent the mean ± standard deviation (SD).

The concentration dependence at 0.5 µM, 1 µM, 2µM, and 5 µM of the selected peptides *Dabcyl*-RR-azaGly-RRK(*Cf*), *Dabcyl*-azaGly-RRRRK(*Cf*), and *Dabcyl*-RRRR-azaGly-K(*Cf*) were studied. Cellular uptake was measured in the A-431 cell line at 37 °C for 90 min (Figure 4). All the relative fluorescence intensities were calculated based on the fluorescence intensity of Cf-Arg_8_ at 5 μM (100%). The data demonstrated that all azaGly-containing peptides have enhanced internalization when the concentration was increased between 0.5 µM and 5 µM. *Dabcyl*-RR-azaGly-RRK(*Cf*) and *Dabcyl*-azaGly-RRRRK(*Cf*) were the best, while the *Dabcyl*-RRRR-azaGly-K(*Cf*) peptide had significantly lower internalization. Between 0.5 and 2 µM, the two best peptides had a similar cellular uptake to the octaarginine.

### 3.3. The Mechanism of Internalization

**Figure 5:**
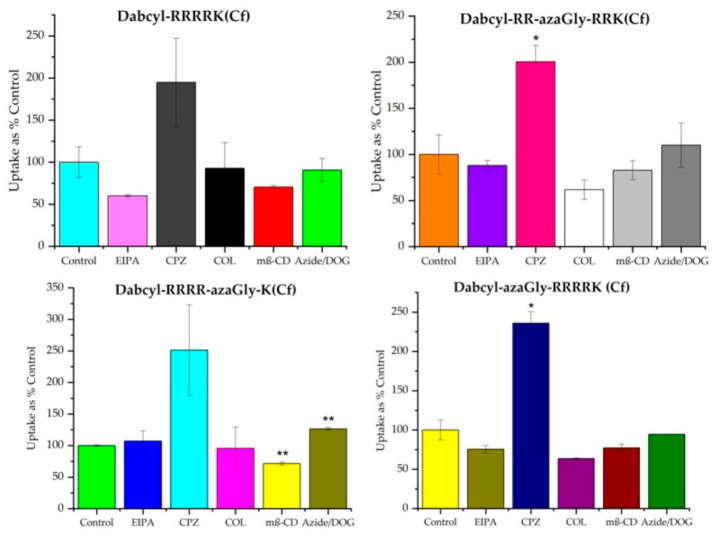
The role of different endocytic routes in the internalization of peptides into A-431 cells was studied after pretreating the cells with EIPA (50 µM), CPZ (30 µM), mβ-CD (2.5 mM), COL (10 mM), NaN_3_ (50 µM), and DOG (25 mM) for 30 min, followed by treatment with peptides (5 µM) for 90 min. Any significant difference from the control was determined by Student’s *t*-test (* *p* < 0.05). Data represent the mean *±* standard deviation (SD). Significant difference from the control was measured using Student’s *t*-test (* *p* < 0.05, ** *p* < 0.01).

The cellular uptake mechanisms of selected peptides were studied on the A-431 cell line. Their routes are compared to the reference peptide *Dabcyl*-RRRRK(*Cf*). In these experiments, various inhibitors were applied: chlorpromazine (CPZ) as an inhibitor of clathrin-mediated endocytosis [38,40], 5-(N-ethyl-N-isopropyl) amiloride (EIPA) as micropinocytosis inhibitor [39], colchicine (COL) to inhibit microtubule formation [41], and methyl-beta-cyclodextrin (CyD) to inhibit caveolae-mediated endocytosis [42]. In addition, A-431 cells were pretreated with sodium azide (NaN_3_) and 2-deoxyglucose (DOG), resulting in depleted ATP, and therefore the energy-dependent pathways are inhibited (Figure 5).

The NaN_3_/DOG treatment did not reduce cellular peptide uptake, demonstrating that ATP depletion is overridden by other mechanisms. However, the inhibition of some energy-dependent processes selectively may reduce the internalization of peptides. The effect of different inhibitors was dependent on the sequence of peptides. The only exception was the effect of CPZ, which increased the cellular uptake of all peptides. The internalization of Dabcyl-modified tetraarginine (*Dabcyl*-RRRRK(*Cf*)) was inhibited by EIPA and mβ-CD. The cellular uptake of peptides with azaGly at the N-terminus and at the middle of the sequence was also inhibited by EIPA and mβ-CD (*Dabcyl*-RR-azaGly-RRK(*Cf*), 12% and 17%; *Dabcyl*-azaGly-RRRRK(*Cf*), 25% and 23%, respectively), but the COL showed inhibitory activity too (*Dabcyl*-RR-azaGly-RRK(*Cf*), 38%; *Dabcyl*-azaGly-RRRRK(*Cf*), 36%). The internalization of the peptide *Dabcyl*-RRRR-azaGly-K(*Cf*) was different from the other two azaGly-containing peptides. Its cellular uptake was inhibited only by mβ-CD.

## 4. Discussion

The family of CPPs as a promising drug delivery moiety has attracted considerable interest and induced much research. These short peptides can be effectively used to deliver a wide range of cargo into different cells. Although there are many successful demonstrations of their usage as delivery units, the effort to improve their internalization, to understand their structure–activity relationship, and to achieve cell or cell organelle specificity has not decreased. It was proven earlier that often a small chemical modification may cause a dramatic change in cellular uptake, and therefore many kinds of modifications are studied. These modifications can aim to improve cellular uptake, increase half-life, or enhance stability. The simplest method is to introduce unnatural amino acids into the sequence [56].

In our research group, we also used several types of unnatural amino acids to improve the performance or alter the internalization mechanism of well-known (penetratin) or very ineffective (tetraarginine) CPPs [22,27,30]. One of them was aza-glycine [30]. This modification of a peptide seems minimal—only the C^α^-atom of one or more amino acid residue(s) is replaced by an N-atom—yet it can cause dramatic changes in the peptide structure and activity [30]. Despite numerous successful applications, this modification has not really caught on. One explanation could be that there are no aza-amino acids that can be bought and coupled as easily as natural amino acids. Its use needs the formation of an aza-amino acid on the resin during synthesis, and the efficiency of this synthesis is very poor in many cases [29]. The application of aza-glycine to change the structure–activity relationship is very convenient because common reagents should be used for the synthesis (Fmoc-NH-NH_2_ and CDI), and this reaction has a high yield. Although the missing side-chain may reduce or diminish the biological activity, the altered structure caused by the aza-amino acid backbone may compensate for this effect and can give us important insight on the structure–activity relationship. We noticed this when Trp residue or residues were replaced by aza-glycine in penetratin [30]. The aza-glycine-modified penetratin had the same internalization ability despite the missing side chain of the Trp residue. The important role of this Trp residue in the cellular uptake of penetratin was proven earlier [10,44]. One of our effective CPP families contains the Dabcyl group and an aromatic ring in the side chain (*Dabcyl*-RR-W-RRK(*Cf*)) [27] or in the backbone (*Dabcyl*-PABA-RRRRK(*Cf*)) [22]. Without this aromatic moiety, the *Dabcyl*-RRRRK(*Cf*) showed very weak internalization [16,22,27].

Based on these findings, we studied the effect of aza-glycine substitution on the internalization of Dabcyl-containing tetraarginine derivatives. Derivatives with azaGly at the C- and N-terminus and in the middle of the peptide were synthesized (Table 1). As peptides with two Trp residues had good internalization, their azaGly-modified derivatives were examined too (Table 1). When one azaGly was inserted into the sequence, the peptides had better cellular uptake than the Dabcyl-containing tetraarginine, but they were less active than the *Dabcyl*-RR-W-RRK(*Cf*) (Figure 2). The position of azaGly did not have any influence on the cellular uptake of peptides (Figure 2). Our results show that, in the case of these short peptides, the aromatic side-chain of the Trp residue makes a high contribution to the cellular uptake; without this, the efficiency of internalization is drastically decreased. The results from this study and an earlier one also demonstrated that the position of Trp has a high impact on the efficiency of peptides. It suggests that not only the interaction with the membrane (it should be the same independent of the position) but the arrangement of side-chains also has an effect on the internalization. The missing side-chain of azaGly means that the increased internalization, which results in a twice-higher cellular uptake than that of tetraarginine, does not come from the additional interaction, but from the structural changes induced by the modification of the peptide backbone. Tetraarginine derivatives with two Trp residues have a lower cellular uptake than *Dabcyl*-RR-W-RRK(*Cf*), but they are better than the tetraarginine (Figure 3). In these peptides, besides the Trp in the middle of the peptide, the position of the additional Trp was varied. The peptide with two Trp residues in the middle was the best, although its azaGly analog was the weakest CPP. In the other case, there was no difference between the Trp- and azaGly-containing derivatives (Figure 3). It seems that the increased hydrophobicity or aromaticity in the middle of the sequence has an additional effect that cannot be replaced by the two azaGly-caused structural changes. But, in the other cases, azaGly can be used without any reduction in the cellular uptake. When the concentration dependence of cellular uptake was examined, octaarginine, as a very efficient CPP, was applied too (Figure 4). Derivatives with one azaGly were close to the octaarginine in efficiency at the highest concentration used, and they had the same cellular uptake at lower concentrations. These results demonstrated the usefulness of azaGly substitution to change the activity of peptides and, in this case, to enhance their cellular uptake.

When chemical modification(s) are used to improve a CPP, the extent of the cellular uptake is only one aspect. Many times, the altered internalization mechanism is also desired. In many CPPs, the main mechanism of internalization relies on some form of endocytosis, resulting in endosome-entrapped cargo. This jeopardizes the biological activity of cargo. Using different inhibitors, the mechanism of internalization of azaGly-modified peptides was studied. We were interested in whether azaGly introduction may alter the internalization pathway of the original peptide (*Dabcyl*-RRRRK(*Cf*)). In the case of the three azaGly-containing peptides, the picture was different, and none of them were similar to the original peptide. The effect of the inhibitors depended on the position of azaGly. The effect of inhibitors varied depending on the position of azaGly, indicating that its location within the peptide sequence modulates the contribution of different internalization routes. Peptides with azaGly at the N-terminus or in the middle of the sequence were sensitive to EIPA, mβ-CD, and colchicine, suggesting uptake through macropinocytosis, caveolae-mediated endocytosis, and microtubule-dependent processes. In contrast, azaGly at the C-terminal position limited uptake predominantly to caveolae-mediated endocytosis. The changed patterns of the effect of inhibitors indicate the altered entry mechanism caused by the azaGly substitution. Overall, these findings suggest that azaGly can fine-tune the internalization pathway without compromising cellular uptake, although the exact mechanisms of internalization have not yet been fully elucidated. Further experiments are necessary to get more precise insights into the cellular entry.

Based on these observations, we would like to gain insight into the effect of azaGly modification on the structure of the peptide. Thus, two different calculations were made to reveal the influence of this modification on the peptide structure.

**Figure 6:**
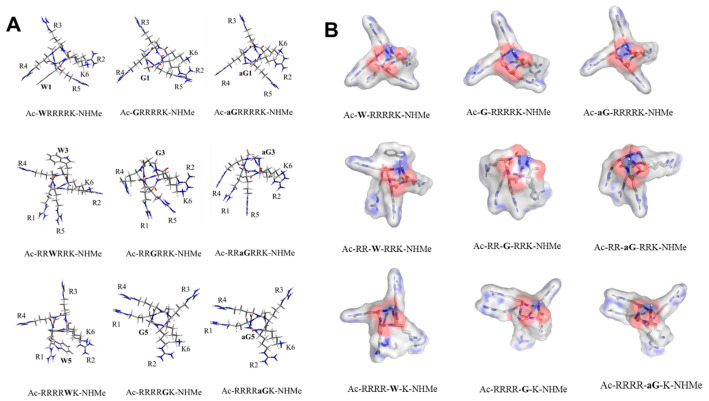
SMD/LC-ωPBE-D3/3-21+G(d)-optimized structures of model peptides **1–9** in water (**A**) and the surface model of peptides (**B**). The respective figures were generated using GaussView and PyMol. The blue, red, gray colors represent the nitrogen, oxygen, and carbon atoms, respectively.

To examine the impact of azaGly residue on the structural properties of peptides, we selected nine model peptides, shown in Figure 6. The N-terminal Trp^1^ residue was replaced by Gly or azaGly (peptides **1–3**); the Trp^3^ was replaced by Gly or azaGly (peptides **4–6**); or the C-terminal Trp^5^ was replaced by Gly or azaGly (peptides **7–9**). The Pep-Fold4 server was used to generate the starting structures of peptides **1–9.** These starting structures were fully optimized in water using SMD/LC-ωPBE-D3/3-21+G(d) level of theory (Figure 6A and SI Figure S28). The structures of peptides adopted the α-helix as expected (Figure S28). Note that the shape of peptides is different depending on the position of Trp (peptides **1**, **4**, and **7**). This is the case for the positions of Gly (peptides **2**, **5**, and **8**) and azaGly (peptides **3**, **6**, and **9**) (Figure 6B). The surfaces of Trp-containing peptides are different from those of azaGly-containing peptides, as expected. Interestingly, the surfaces of the Gly- and azaGly-inserted peptides are similar if the position of the Gly and azaGly residue is the same (Figure 6B). The different surfaces of the peptide are related to the orientation of hydrophilic residues such as Arg and Lys. For example, Arg^2^, Arg^5^, and Lys^6^ are closed for peptides **1–3**; (Arg^1^ and Arg^5^) and (Arg^2^ and Lys^6^) are closed for peptides **4–6**; (Arg^1^ and Arg^4^); and (Arg^2^ and Lys^6^) are closed for peptides **8** and **9** (Figure 6). These structural features imply that model peptides have different amphiphilicity. Thus, the structural changes in peptides may be related to the different cellular uptake capabilities of peptides. We hypothesize that peptide modification may alter hydrophobic moments and the binding affinity of peptides to the surface of a negatively charged membrane.

To understand the cellular uptake ability of the synthesized peptides in Figure 2, Figure 3 and Figure 4, the sensing and binding free energy difference (kJ/mol) of model peptides, as shown in Table 2, with a negatively charged membrane (POPC/POPG) was obtained using the PMIpred server (assessed on 8 January 2026), which is using a neural network model trained on molecular dynamics simulation data [50,51]. PMIpred predicted the hydrophobicity, hydrophobic moment, and sensing and binding free energy difference (ΔF_sm (R = 50)) of model peptides to the negatively charged membrane (Table 2).

We classified three groups (Group I is the model peptides shown in Figure 2; Group II is the models shown in Figure 3; Group III is the models shown in Figure 4) (Table 2). Because PMIpred did not provide the binding free energy of a non-standard peptide at the membrane surface, we used Gly-containing peptides instead of azaGly-containing peptides. We assumed that Gly-containing peptides and azaGly-containing peptides have similar binding capabilities to the membrane because of their similar surface properties (Figure 6). We expect these parameters to be useful for identifying trends in the internalization of the peptides discussed in this work.

**Table 2:** The predicted sensing and binding free energy (kJ/mol) from a peptide sequence using PMIpred.

**Group I**	**Hydrophobicity**	**Hydrophobic Moment**	**ΔΔF**	**ΔF_sm** **(R = 50) ^a^**	**Relative Fluorescence (%) ^b^**
ARRWRRKA	−0.27	0.574	−2.764	−55.361	100
ARRGRRKA	−0.55	0.315	−2.016	−42.191	45
ARRRRWKA	−0.27	0.242	−3.158	−62.304	70
ARRRRGKA	−0.55	0.125	−2.021	−42.284	30
AGRRRRKA	−0.55	0.210	−1.847	−39.206	30
ARRRRKA	−0.63	0.219	−2.060	−52.481	12
**Group II**	**Hydrophobicity**	**Hydrophobic Moment**	**ΔΔF**	**ΔF_sm** **(R = 50)**	**Relative Fluorescence (%) ^b^**
ARRWWRRKA	0.01	0.522	−3.367	−55.784	100
ARRGGRRKA	−0.49	0.206	−1.859	−33.786	37
AWRRWRRKA	0.01	0.696	−4.726	−75.614	63
AGRRGRRKA	−0.49	0.265	−1.724	−31.824	57
ARRWRRWKA	0.01	0.581	−3.557	−58.552	53
ARRGRRGKA	−0.59	0.166	−1.826	−38.846	41
ARRRRKA	−0.63	0.219	−2.060	−52.481	21
**Group III**	**Hydrophobicity**	**Hydrophobic Moment**	**ΔΔF**	**ΔF_sm** **(R = 50)**	**Relative Fluorescence (%) ^b^**
AGRRRRKA	−0.55	0.210	−1.847	−39.206	60
ARRGRRKA	−0.55	0.315	−2.016	−42.191	72
ARRRRGKA	−0.55	0.125	−2.021	−42.284	50
ARRRRRRRRA	−0.75	0.085	−1.715	−39.391	100

^a.^ Negatively charged membrane (POPC/POPG) ^b.^ The values for FITC means used those of the azaGly-containing peptides in Figure 2, Figure 3 and Figure 4.

Group I: ARRWRRKA (relative fluorescence = 100%) has the highest hydrophobic moment (0.574) and a moderately high ΔΔF (−2.764 kJ/mol) and ΔF_sm (R = 50) value (−55.361 kJ/mol). ARRRRWKA (relative fluorescence = 70%) also has the highest ΔF_sm (R = 50) value but has a moderate hydrophobic moment (0.242). The Gly residue-inserted peptides, A**G**RRRRKA, ARR**G**RRKA, and ARRRK**G**A, showed low relative fluorescence (30–45%) and have a low hydrophobic moment and a low ΔF_sm (R = 50) value. The results suggest that the cellular uptakes of peptides may be determined by the combination of the hydrophobic moment and the binding energy of peptides to the surface of a negatively charged membrane. Compared to ARRRRWKA and ARRRRRKA, both peptides showed strong binding to the membrane, but the relative fluorescence declines (70% to 12%). The results suggest that the excessive charge without optimal amphiphilicity reduces uptake despite favorable free-energy terms.

Group II: Peptides with a higher hydrophobic moment (ARRWWRRKA, AWRRWRRKA, and ARRWRRWKA) resulted in a high relative fluorescence intensity (53–100%). In the case of peptides in which the Trp residues are separated (AWRRWRRKA and ARRWRRWKA), the amphiphilicity correlates positively with cellular uptake (Supplementary Materials Figure S29). The hydrophobic moment of peptide ARRWWRRKA is not the highest, but it has the most pronounced internalization. Its membrane binding energy is less than that of the two other peptides (AWRRWRRKA and ARRWRRWKA). This may indicate that peptides could stick in the membrane because of the too strong membrane interaction, and this effect may reduce their internalization. The introduction of Gly in peptides (ARRGGRRKA, AGRRGRRKA, and ARRGRRGKA) reduced the hydrophobic moment and the binding free energy to the surface of the membrane, resulting in a reduced relative fluorescence (37–41%). The results indicate that introducing Gly between Arg clusters may disrupt the contiguous cationic face needed for efficient interaction with the membrane. The ARRRKA peptide shows a strong interaction with the membrane, but other factors, such as structural features or peptide length, may influence cellular uptake in group II.

Group III: Depending on the position of the Gly residue in model peptides (AGRRRRKA, ARRGRRKA, ARRRRGKA), the hydrophobic moment of the peptides is changed. The highest hydrophobic moment (0.315) of ARRGRRKA shows a high relative fluorescence (70%). However, the AR_8_A peptide has the lowest hydrophobic moment (0.085), showing the highest relative fluorescence compared with the other peptides. This may indicate, in very Arg-rich contexts, that the net charge and linearity of Arg residues can sometimes dominate over weaker amphiphilicity. It may also show that octaarginine has a different membrane interaction pattern and mode of membrane penetration than peptides with more or less hydrophobic amino acid residues. When the modification of the hydrophobicity of a peptide results in increased internalization, the effect of self-assembly should be taken into account. Rationally designed CPPs have been shown to act as building blocks for self-assembled nanostructures with effective cellular uptake [57,58]. The insertion of a hydrophobic Dabcyl group may promote the self-assembly of peptides, thus increasing the cellular uptake.

Based on our knowledge, this is the first study to examine the effect of an aza-amino acid substitution on the cellular uptake of oligoarginines. Our results suggest that aza-glycine substitution represents a valuable strategy to fine-tune peptide properties for improved cellular delivery while maintaining high activity at low concentrations.

## Figures and Tables

**Table 1:** Chemical characterization of the peptides.

Sequence	^a^ R_t_	M _calc_	^b^ M _meas_
*Dabcyl*-RR**W**RRK(*Cf*)	14.9	1564.7	1564.8
*Dabcyl-*RR-**azaGly**-RRK(*Cf*)	13.9	1436.7	1436.8
*Dabcyl*-RRRR**W**K(*Cf*)	15.4	1564.7	1565.7
*Dabcyl*-RRRR-**azaGly**-K(*Cf*)	15.6	1436.7	1436. 6
*Dabcyl*-**azaGly**-RRRRK(*Cf*)	15.0	1436.7	1436.7
*Dabcyl*-RR**WW**RRK(*Cf*)	18.2	1750.8	1751.8
*Dabcyl*-RR-**azaGly**-**azaGly**-RRK(*Cf*)	16.4	1494.7	1494.6
*Dabcyl*-RR**W**RR**W**K(*Cf*)	17.1	1750.8	1750.8
*Dabcyl*-RR-**azaGly**-RR-**azaGly**-K(*Cf*)	16.4	1494.7	1494.5
*Dabcyl*-**W**RR**W**RRK(*Cf*)	17.6	1750.8	1751.0
*Dabcyl*-**azaGly**-RR-**azaGly**-RRK(*Cf*)	17.3	1494.7	1494.7
*Cf*-R_8_	12.5	1623.9	1623.8
*Dabcyl*-RRRRK(*Cf*)	12.3	1378.7	1379.6

^a^ The analytical chromatogram was obtained using a Hypersil Hypurity C18 column (4.6 mm × 150 mm, 5 μm,190 A). Linear gradient elution (0 min 0% B, 2 min 0% B, 22 min 90% B) was used at a 1 mL/min flow rate. The absorbance was measured at λ = 220 nm. ^b^ The mass spectrometric analysis was conducted on a Bruker Amazon SL (Bremen, Germany). The samples were dissolved in acetonitrile–water (50:50, *v*/*v*), containing 0.1% formic acid.

## Data Availability

Data are contained within the article and Supplementary Materials.

## Supplementary Materials

The following supporting information can be downloaded at: https://www.mdpi.com/article/10.3390/biomedicines14051025/s1. Figures S1–S13: HPLC chromatograms of compounds; Figures S14–S26: MS spectra of compounds; Figure S27: Cytotoxicity of the peptides; Figure S28: SMD/LC-ωPBE-D3/3-21+G*-optimized structure of model peptides **1**–**9**; Figure S29: Hydrophobic moment vs FITC means for model peptides; Figure S30: Representative histograms and dot plots; Figures S31 and S32: FITC mean values of cellular uptake.

## References

[1] Ghosh P., Raj N., Verma H., Patel M., Chakraborti S., Khatri B., Doreswamy C.M., Anandakumar S.R., Seekallu S., Dinesh M.B. (2023). An Amide to Thioamide Substitution Improves the Permeability and Bioavailability of Macrocyclic Peptides. Nat. Commun..

[2] Szabó I., Yousef M., Soltész D., Bató C., Mező G., Bánóczi Z. (2022). Redesigning of Cell-Penetrating Peptides to Improve Their Efficacy as a Drug Delivery System. Pharmaceutics.

[3] Bánóczi Z., Alexa A., Farkas A., Friedrich P., Hudecz F. (2008). Novel Cell-Penetrating Calpain Substrate. Bioconjug. Chem..

[4] Alexa A., Ember O., Szabó I., Mo’ath Y., Póti Á.L., Reményi A., Bánóczi Z. (2021). Peptide Based Inhibitors of Protein Binding to the Mitogen-Activated Protein Kinase Docking Groove. Front. Mol. Biosci..

[5] Futaki S., Arafiles J.V.V., Hirose H. (2020). Peptide-Assisted Intracellular Delivery of Biomacromolecules. Chem. Lett..

[6] Bánóczi Z., Keglevich A., Szabó I., Ranđelović I., Hegedüs Z., Regenbach F.L., Keglevich P., Lengyel Z., Gorka-Kereskényi Á., Dubrovay Z. (2018). The Effect of Conjugation on Antitumor Activity of Vindoline Derivatives with Octaarginine, a Cell-Penetrating Peptide. J. Pept. Sci..

[7] McClorey G., Banerjee S. (2018). Cell-Penetrating Peptides to Enhance Delivery of Oligonucleotide-Based Therapeutics. Biomedicines.

[8] Green M., Loewenstein P.M. (1988). Autonomous Functional Domains of Chemically Synthesized Human Immunodeficiency Virus Tat Trans-Activator Protein. Cell.

[9] Vivès E., Brodin P., Lebleu B. (1997). A Truncated HIV-1 Tat Protein Basic Domain Rapidly Translocates through the Plasma Membrane and Accumulates in the Cell Nucleus. J. Biol. Chem..

[10] Derossi D., Joliot A.H., Chassaing G., Prochiantz A. (1994). The Third Helix of the Antennapedia Homeodomain Translocates through Biological Membranes. J. Biol. Chem..

[11] Frankel A.D., Pabo C.O. (1988). Cellular Uptake of the Tat Protein from Human Immunodeficiency Virus. Cell.

[12] Ziegler A., Nervi P., Dürrenberger M., Seelig J. (2005). The Cationic Cell-Penetrating Peptide CPPTAT Derived from the HIV-1 Protein TAT Is Rapidly Transported into Living Fibroblasts: Optical, Biophysical, and Metabolic Evidence. Biochemistry.

[13] Schmidt N., Mishra A., Lai G.H., Wong G.C.L. (2010). Arginine-Rich Cell-Penetrating Peptides. FEBS Lett..

[14] Mitchell D.J., Kim D.T., Steinman L., Fathman C.G., Rothbard J.B. (2000). Polyarginine Enters Cells More Efficiently than Other Polycationic Homopolymers. J. Pept. Res..

[15] Schug K.A., Lindner W. (2005). Noncovalent Binding between Guanidinium and Anionic Groups: Focus on Biological- and Synthetic-Based Arginine/Guanidinium Interactions with Phosph [on] Ate and Sulf [on] Ate Residues. Chem. Rev..

[16] Szabó I., Illien F., Dókus L.E., Yousef M., Baranyai Z., Bősze S., Ise S., Kawano K., Sagan S., Futaki S. (2021). Influence of the Dabcyl Group on the Cellular Uptake of Cationic Peptides: Short Oligoarginines as Efficient Cell-Penetrating Peptides. Amino Acids.

[17] Szabó I., Orbán E., Schlosser G., Hudecz F., Bánóczi Z. (2016). Cell-Penetrating Conjugates of Pentaglutamylated Methotrexate as Potential Anticancer Drugs against Resistant Tumor Cells. Eur. J. Med. Chem..

[18] DeRonde B.M., Birke A., Tew G.N. (2015). Design of Aromatic-Containing Cell-Penetrating Peptide Mimics with Structurally Modified π Electronics. Chem. A Eur. J..

[19] Swiecicki J.M., Di Pisa M., Lippi F., Chwetzoff S., Mansuy C., Trugnan G., Chassaing G., Lavielle S., Burlina F. (2015). Unsaturated Acyl Chains Dramatically Enhanced Cellular Uptake by Direct Translocation of a Minimalist Oligo-Arginine Lipopeptide. Chem. Commun..

[20] Qian Z., Liu T., Liu Y.Y., Briesewitz R., Barrios A.M., Jhiang S.M., Pei D. (2013). Efficient Delivery of Cyclic Peptides into Mammalian Cells with Short Sequence Motifs. ACS Chem. Biol..

[21] Song J., Qian Z., Sahni A., Chen K., Pei D. (2019). Cyclic Cell-Penetrating Peptides with Single Hydrophobic Groups. ChemBioChem.

[22] Yousef M., Szabó I., Murányi J., Illien F., Soltész D., Bató C., Tóth G., Batta G., Nagy P., Sagan S. (2022). Cell-Penetrating Dabcyl-Containing Tetraarginines with Backbone Aromatics as Uptake Enhancers. Pharmaceutics.

[23] Shirani A., Mojarrad J.S., Farkhani S.M., Khosroshahi A.Y., Zakeri-Milani P., Samadi N., Sharifi S., Mohammadi S., Valizadeh H. (2015). The Relation between Thermodynamic and Structural Properties and Cellular Uptake of Peptides Containing Tryptophan and Arginine. Adv. Pharm. Bull..

[24] Tompa P., Buzder-Lantos P., Tantos A., Farkas A., Szilágyi A., Bánóczi Z., Hudecz F., Friedrich P. (2004). On the Sequential Determinants of Calpain Cleavage. J. Biol. Chem..

[25] Roloff A., Nelles D.A., Thompson M.P., Yeo G.W., Gianneschi N.C. (2018). Self-Transfecting Micellar RNA: Modulating Nanoparticle Cell Interactions via High Density Display of Small Molecule Ligands on Micelle Coronas. Bioconjug. Chem..

[26] Mandal S., Mann G., Satish G., Brik A. (2021). Enhanced Live-Cell Delivery of Synthetic Proteins Assisted by Cell-Penetrating Peptides Fused to DABCYL. Angew. Chem. Int. Ed..

[27] Yousef M., Szabó I., Biri-Kovács B., Szeder B., Illien F., Sagan S., Bánóczi Z. (2021). Modification of Short Non-Permeable Peptides to Increase Cellular Uptake and Cytostatic Activity of Their Conjugates. ChemistrySelect.

[28] Bató C., Szabó I., Bánóczi Z. (2023). Enhancing Cell Penetration Efficiency of Cyclic Oligoarginines Using Rigid Scaffolds. Pharmaceutics.

[29] Tarchoun K., Yousef M., Bánóczi Z. (2022). Azapeptides as an Efficient Tool to Improve the Activity of Biologically Effective Peptides. Future Pharmacol..

[30] Tarchoun K., Soltész D., Farkas V., Lee H.J., Szabó I., Bánóczi Z. (2024). Influence of Aza-Glycine Substitution on the Internalization of Penetratin. Pharmaceutics.

[31] Lee H.J., Ahn I.A., Ro S., Choi K.H., Choi Y.S., Lee K.B. (2000). Role of Azaamino Acid Residue in β-Turn Formation and Stability in Designed Peptide. J. Pept. Res..

[32] Zouikri M., Vicherat A., Aubry A., Marraud M., Boussard G. (1998). Azaproline as a Beta-Turn-Inducer Residue Opposed to Proline. J. Pept. Res..

[33] Zhang W.J., Berglund A., Kao J.L.F., Couty J.P., Gershengorn M.C., Marshall G.R. (2003). Impact of Azaproline on Amide Cis-Trans Isomerism: Conformational Analyses and NMR Studies of Model Peptides Including TRH Analogues. J. Am. Chem. Soc..

[34] Lee H.J., Liu S.W., Sulyok-Eiler M., Harmat V., Farkas V., Bánóczi Z., El Khabchi M., Shawn Fan H.J., Hirao K., Song J.W. (2024). Neighbor Effect on Conformational Spaces of Alanine Residue in Azapeptides. Heliyon.

[35] Lecoq A., Boussard G., Marraud M., Aubry A. (1993). Crystal State Conformation of Three Azapeptides Containing the Azaproline Residue, a β-Turn Regulator. Biopolymers.

[36] André F., Boussard G., Bayeul D., Didierjean C., Aubry A., Marraud M. (1997). Aza-Peptides. II. X-Ray Structures of Aza-Alanine and Aza-Asparagine-Containing Peptides. J. Pept. Res..

[37] Gibson C., Goodman S.L., Hahn D., Hölzemann G., Kessler H. (1999). Novel Solid-Phase Synthesis of Azapeptides and Azapeptoides via Fmoc- Strategy and Its Application in the Synthesis of RGD-Mimetics. J. Org. Chem..

[38] Wieczerzak E., Drabik P., Łankiewicz L., Ołdziej S., Grzonka Z., Abrahamson M., Grubb A., Brömme D. (2002). Azapeptides Structurally Based upon Inhibitory Sites of Cystatins as Potent and Selective Inhibitors of Cysteine Proteases. J. Med. Chem..

[39] Koivusalo M., Welch C., Hayashi H., Scott C.C., Kim M., Alexander T., Touret N., Hahn K.M., Grinstein S. (2010). Amiloride Inhibits Macropinocytosis by Lowering Submembranous PH and Preventing Rac1 and Cdc42 Signaling. J. Cell Biol..

[40] Gomes dos Reis L., Lee W.H., Svolos M., Moir L.M., Jaber R., Engel A., Windhab N., Young P.M., Traini D. (2020). Delivery of PDNA to Lung Epithelial Cells Using PLGA Nanoparticles Formulated with a Cell-Penetrating Peptide: Understanding the Intracellular Fate. Drug Dev. Ind. Pharm..

[41] Starling D., Duncan R., Lloyd J.B. (1983). The Role of Microtubules in Pinocytosis. Inhibition of Fluid-Phase Pinocytosis in the Rat Visceral Yolk Sac by Mitoclasic and Related Agents. Cell Biol. Int. Rep..

[42] Fittipaldi A., Ferrari A., Zoppé M., Arcangeli C., Pellegrini V., Beltram F., Giacca M. (2003). Cell Membrane Lipid Rafts Mediate Caveolar Endocytosis of HIV-1 Tat Fusion Proteins. J. Biol. Chem..

[43] RPBS Web Portal. https://mobyle2.rpbs.univ-paris-diderot.fr/cgi-bin/portal.py#forms::PEP-FOLD4.

[44] Frisch M.J., Trucks G.W., Schlegel H.B., Scuseria G.E., Robb M.A., Cheeseman J.R., Scalmani G., Barone V., Petersson G.A., Nakatsuji H. (2016). Gaussian 16.

[45] Grimme S., Antony J., Ehrlich S., Krieg H. (2010). A Consistent and Accurate Ab Initio Parametrization of Density Functional Dispersion Correction (DFT-D) for the 94 Elements H-Pu. J. Chem. Phys..

[46] Vydrov O.A., Scuseria G.E. (2006). Assessment of a Long-Range Corrected Hybrid Functional. J. Chem. Phys..

[47] Marenich A.V., Cramer C.J., Truhlar D.G. (2009). Universal Solvation Model Based on Solute Electron Density and on a Continuum Model of the Solvent Defined by the Bulk Dielectric Constant and Atomic Surface Tensions. J. Phys. Chem. B.

[48] Dennington R., Keith T.A., Millam J.M. (2016). GaussView.

[49] Schrödinger L., Warren D. (2020). PyMOL.

[50] Van Hilten N., Verwei N., Methorst J., Nase C., Bernatavicius A., Risselada H.J. (2024). PMIpred: A Physics-Informed Web Server for Quantitative Protein–Membrane Interaction Prediction. Bioinformatics.

[51] van Hilten N., Methorst J., Verwei N., Risselada H.J. (2023). Physics-Based Generative Model of Curvature Sensing Peptides; Distinguishing Sensors from Binders. Sci. Adv..

[52] Van Hilten N., Stroh K.S., Risselada H.J. (2022). Efficient Quantification of Lipid Packing Defect Sensing by Amphipathic Peptides: Comparing Martini 2 and 3 with CHARMM36. J. Chem. Theory Comput..

[53] Eisenberg D., Weiss R.M., Terwilliger T.C. (1982). The Helical Hydrophobic Moment: A Measure of the Amphiphilicity of a Helix. Nature.

[54] Fauchere J.-L., Pliska V. (1983). Hydrophobic Parameters Π of Amino Acid Side-Chains from the Partitioning of N-Acetyl-Amino Acid Amides. Eur. J. Med. Chem..

[55] Letoha T., Gaál S., Somlai C., Venkei Z., Glavinas H., Kusz E., Duda E., Czajlik A., Peták F., Penke B. (2005). Investigation of Penetratin Peptides. Part 2. In Vitro Uptake of Penetratin and Two of Its Derivatives. J. Pept. Sci..

[56] Umeno T., Takemoto H., Oba M. (2025). Plasmid DNA Delivery Using Arginine-Rich Cell-Penetrating L/D-Peptides Containing α-Aminoisobutyric Acids. Org. Biomol. Chem..

[57] Shi Y., Lin R., Cui H., Azevedo H.S. (2018). Multifunctional self-assembling peptide-based nanostructures for targeted intracellular delivery: Design, physicochemical characterization, and biological assessment. Biomaterials for Tissue Engineering: Methods and Protocols.

[58] Shi Y., Hu H. (2023). AI accelerated discovery of self-assembling peptides. Biomater. Transl..

