# Supplementary material for: The Effect of Aza-Glycine Substitution on the Internalization of Dabcyl-Containing Short Oligoarginine
Source: Biomedicines. 2026 Apr 30;14(5):1025. doi: 10.3390/biomedicines14051025 (PMC13204268; doi:10.3390/biomedicines14051025)
Supplement: Supplementary file 1 [file biomedicines-14-01025-s001.zip › biomedicines-4233689-supplementary.pdf]

Supplementary Materials

The effect of Aza-Glycine Substitution on the Internalization of Dabcyl-containing short oligoarginine

Karima Tarchoun[1,2], Dóra Soltész[1,2], Ildikó Szabó[3], Jong-Won Song[4], Ho-Jin Lee[5] Zoltán Bánóczi[1,3]

[1] Department of Organic Chemistry, Institute of Chemistry, Faculty of Science, ELTE Eötvös Loránd University, Pázmány Péter Sétány 1/A, 1117 Budapest, Hungary

[2] Hevesy György PhD School of Chemistry, Institute of Chemistry, ELTE Eötvös Loránd University, Pázmány Péter Sétány 1/A, 1117 Budapest, Hungary

[3] HUN-REN-ELTE Research Group of Peptide Chemistry, 1117 Budapest, Hungary

[4] Department of Chemistry Education, Daegu University, Daegudae-ro 201, Gyeongsan-si, Gyeongsangbuk-do, 38453, Republic of Korea (sjoshua@daegu.ac.kr)

[5] Division of Natural and Mathematical Sciences, LeMoyne-Owen College, Memphis, TN, 38126, USA (ho-jin_lee@loc.edu) ORCID: https://orcid.org/0000-0002-3251-4984

1. Materials

The solid support Rink Amide MBHA resin, the Fmoc-protected arginine and tryptophan amino acids, and the compound N, N′- diisopropylcarbodiimide (DIC), were obtained from IRIS BiotechGmbH (Marktredwitz, Germany).The set of chemical compounds thioanisole, 1,2-ethandithiol (EDT), 1.8-diazabicyclo [5.4.0] undec-7-ene (DBU), 1, 1′-carbonyldiimidazole (CDI) and N, N-diisopropylethylamine (DIEA), were purchased from Fluka (Buchs, Switzerland). While, the following inhibitors, Colchicine (COL), Deoxy-D-glucose (DOG), 5-(N-Ethyl-N-isopropyl) amiloride (EIPA), Sodium azide (NaN_3), and the group of reagents, phenol, Ethyl cyano(hydroxyimino)acetate (Oxyma Pure), trifluoroacetic acid(TFA), 5(6)-carboxyfluorescein (Cf), and all other additional chemicals used for biological studies, were sourced from Sigma-Aldrich (Budapest, Hungary) products. Tokyo chemical industries (TCI) provided Fmoc-Hydrazide, methyl-beta-cyclodextrin (CyD), and chlorpromazine (CPZ). 4-((4-(Dimethylamino) phenyl) azo) benzoic acid (Dabcyl) was provided by AAT Bioquest (Pleasanton, CA, USA) . All used solvents for the synthesis, and the purification acquired from Molar Chemicals Ltd (Budapest, Hungary).

1.1.RP-HPLC

The purification and analytical of the crude labelled peptides were performed by the combined system, the analytical RP-HPLC on Exformma (Exformma Technology (ASIA) Co., Ltd, Hong Kong, China),

and the purification on a semi-preparative Phenomenex Jupiter C18 column (250 × 10 mm I.D.) with 10 mm silica (300 Å pore size) (Torrance, CA, USA). In the first system, the used column is Hypersil Hypurity C18 column (4.6 mm × 150 mm, 5 µm, 190 Å).The Linear gradient elution (0 min 0% B; 2 min 0% B; 22 min 90% B) was employed eluent A (0.1% TFA in water) and eluent B (0.1% TFA in acetonitrile-water (80:20, V/V).The flow rate used is 1mL/min, and the detection of peaks take place at λ= 220 nm. All samples were dissolved in small amounts of eluent B and injected into the analytical RP-HPLC. For the purification, all samples were dissolved in eluant A, adding minimum amount of eluant B varied between (10–25% depending on sequence). The flow rate applied is 4 mL/min. Linear gradient elution was utilized, and the peaks were detected at λ= 220 nm.

1.2. Mass spectrometry

The identification of the set of the peptides and the labelled peptides was determined by measuring the molecular mass with ESI-MS using Bruker Amazon SL (Germany). The samples were prepared by dissolving each in water-acetonitrile solution (50:50) with 0.1% formic acid. The prepared samples were centrifuged, followed by direct injection using a syringe pump. The applied parameters were the following: capillary voltage: 4 kV, dry gas: 4 L/min, nebulizer gas: 10 psi, heated capillary temperature: 250 °C.

1.3 Cell Culture

In vitro analysis proceed on A-431 human skin squamous cancer cells (CRL-1555TM). Cells were cultured in Dulbecco's Modified Eagle Medium (DMEM), enriched with 10% heat inactivated foetal calf serum (FCS), L-Glutamine (2 mM), sodium pyruvate (1mM), nonessential amino acids (NEAA), and 1% penicillin-streptomycin (from 10,000 units penicillin and 10 mg/ml streptomycin). Plastic tissue culture flasks were used to maintain the cells under conditions of 37 °C temperature and a humidified atmosphere consisting of 5% CO_2/95% air.

2. Chemical Characterization of Peptides

2.1. RP-HPLC

All labelled peptides were dissolved in a minimum amount of eluent B composed of (0.1% TFA in acetonitrile-water (80:20, v/v), followed by direct injection in the analytical RP-HPLC. This was conducted on Exformma (Exformma Technology (ASIA) Co., Ltd, Hong Kong, China) HPLC system Hypersil Hypurity C18 was used as a column (4.6 mm × 150 mm, 5 µm, 190 Å), and linear gradient elution (0 min 0% B; 2 min 0% B; 22 min 90% B) was applied using eluent A (0.1% TFA in water), and eluent B at a flow rate of 1mL/min. The peaks were detected at wavelength equal to λ= 220 nm.. In several chromatograms, two main peaks appear. Those peaks correspond to two Cf isomers (5-

carboxyfluorescein and 6-carboxyfluorescein), due to the use of a mixture of them as a form of as 5(6)-carboxyfluorescein, during the labelling of the peptides in solution phase within the synthesis.

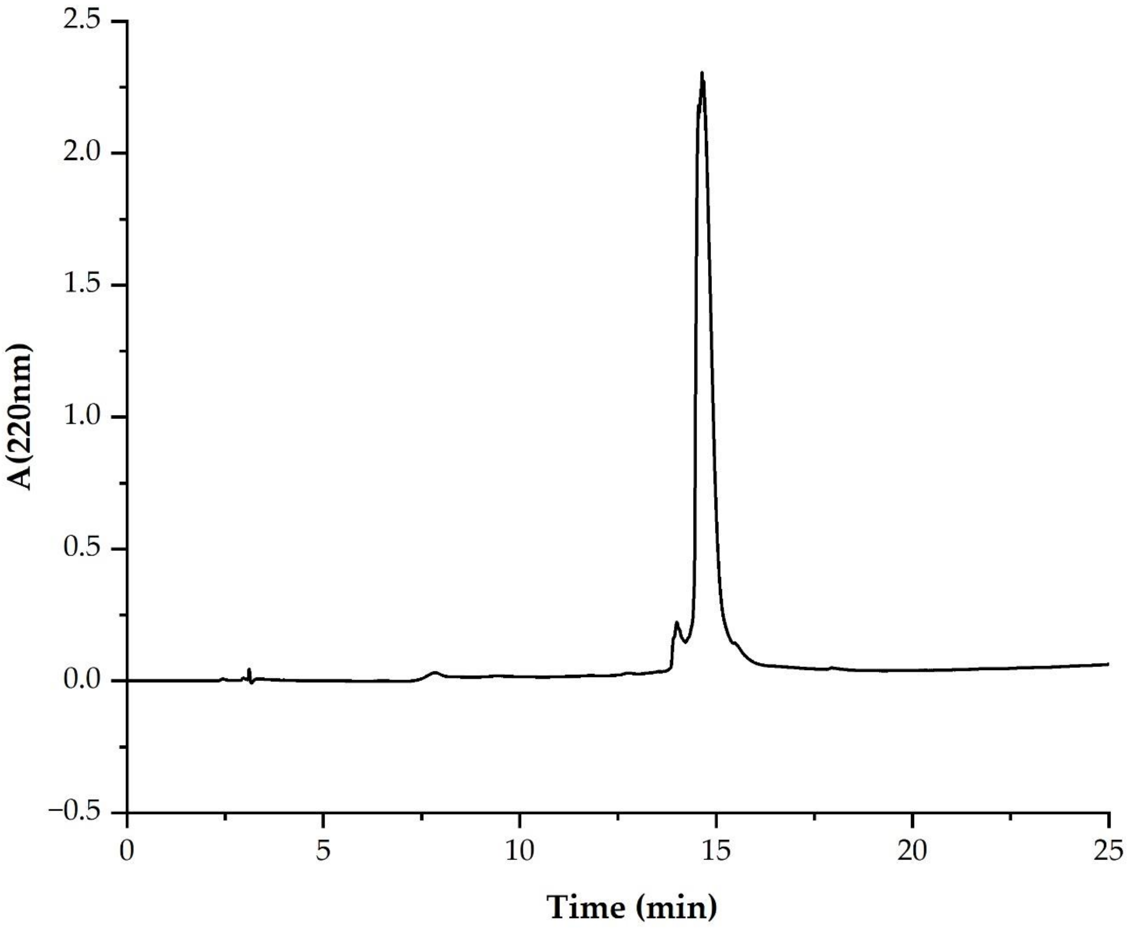

Figure S1. HPLC chromatogram of Dabcyl-RRWRRK(Cf). Retention time was obtained on Hypersil Hypurity C18 column (4.6 mm x 150 mm, 5 µm, 190 Å). The applied linear gradient elution was 0 min 0% B, 2 min 0% B, 22 min 90% B at 1 mL/min flow rate. The detection was carried out at λ = 220 nm. R_t = 14.9 min

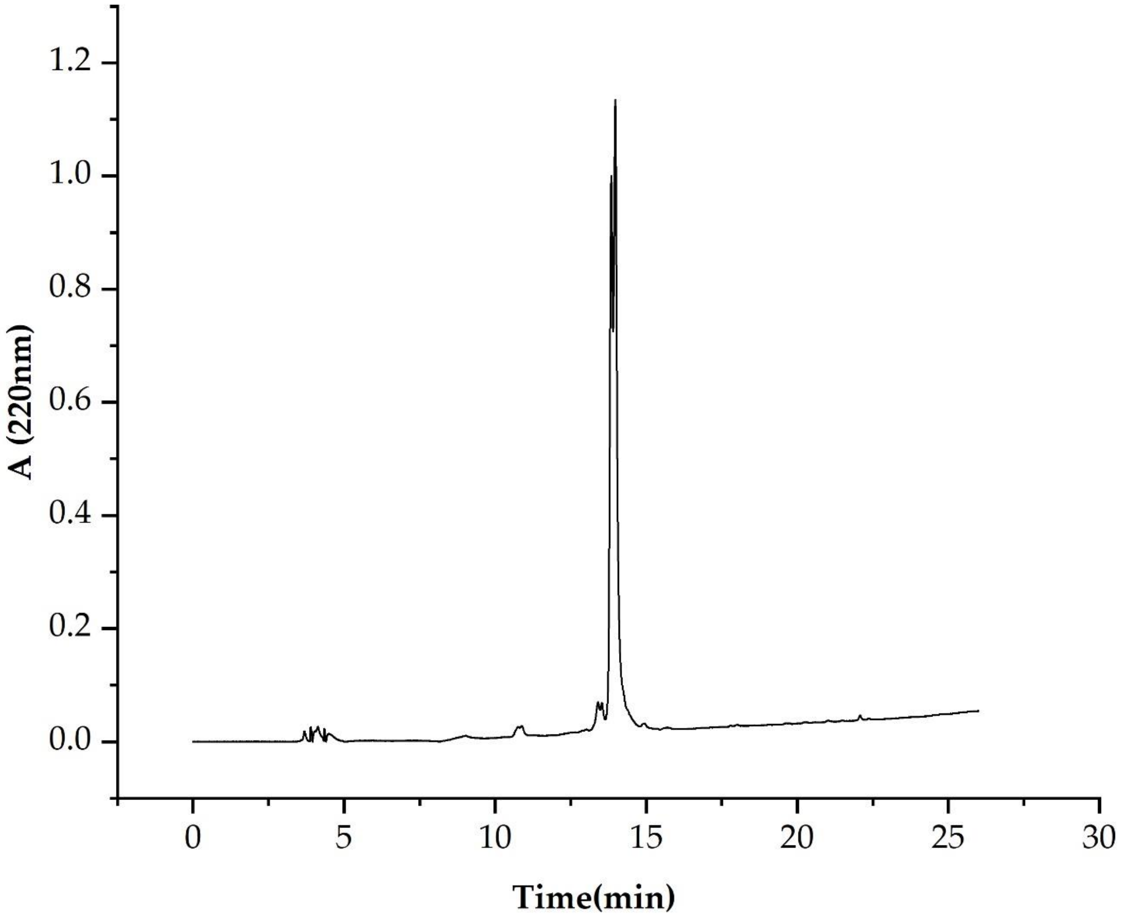

Figure S2. HPLC chromatogram of Dabcyl-RR-azaGly-RRK(Cf). Retention time was obtained on Hypersil Hypurity C18 column (4.6 mm x 150 mm, 5 µm, 190 Å). The applied linear gradient elution was 0 min 0% B, 2 min 0% B, 22 min 90% B at 1 mL/min flow rate. The detection was carried out at λ = 220 nm. R_t = 13.9 min

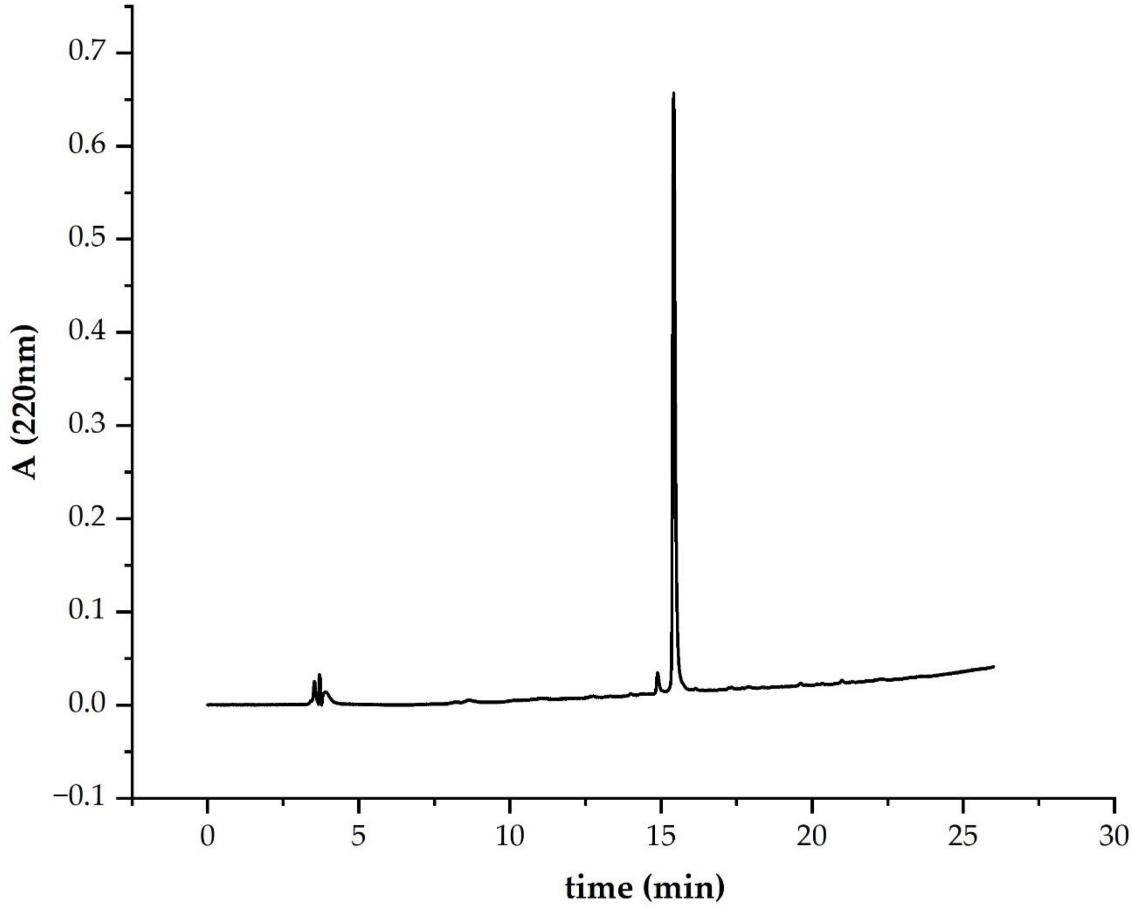

Figure S3. HPLC chromatogram of Dabcyl-RRRRWK(Cf). Retention time was obtained on Hypersil Hypurity C18 column (4.6 mm x 150 mm, 5 µm, 190 Å). The applied linear gradient elution was 0 min 0% B, 2 min 0% B, 22 min 90% B at 1 mL/min flow rate. The detection was carried out at $\lambda = 220$ nm.

$R_t = 15.4$ min

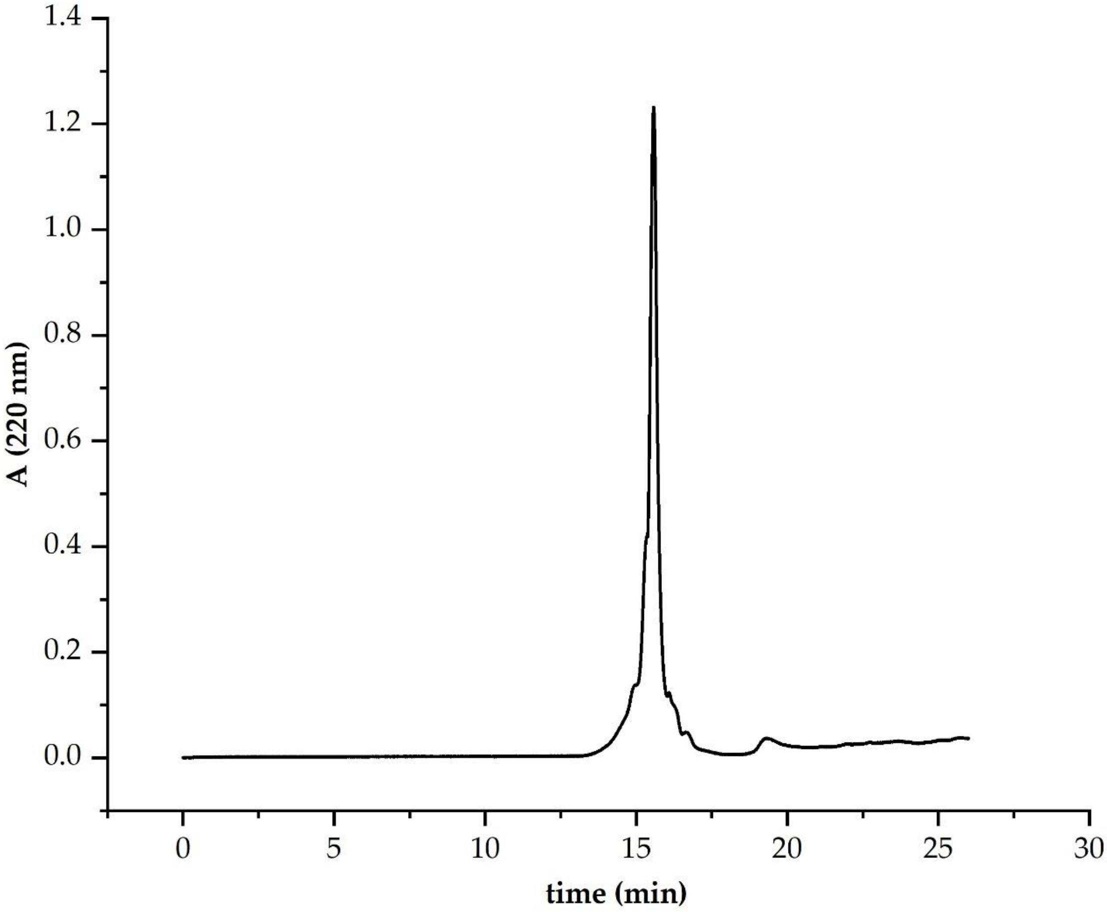

Figure S4. HPLC chromatogram of Dabcyl-RRRR-azaGly-K(Cf). Retention time was obtained on Hypersil Hypurity C18 column (4.6 mm x 150 mm, 5 μm, 190 Å). The applied linear gradient elution was 0 min 0% B, 2 min 0% B, 22 min 90% B at 1 mL/min flow rate. The detection was carried out at λ = 220 nm. R_t = 15.6 min

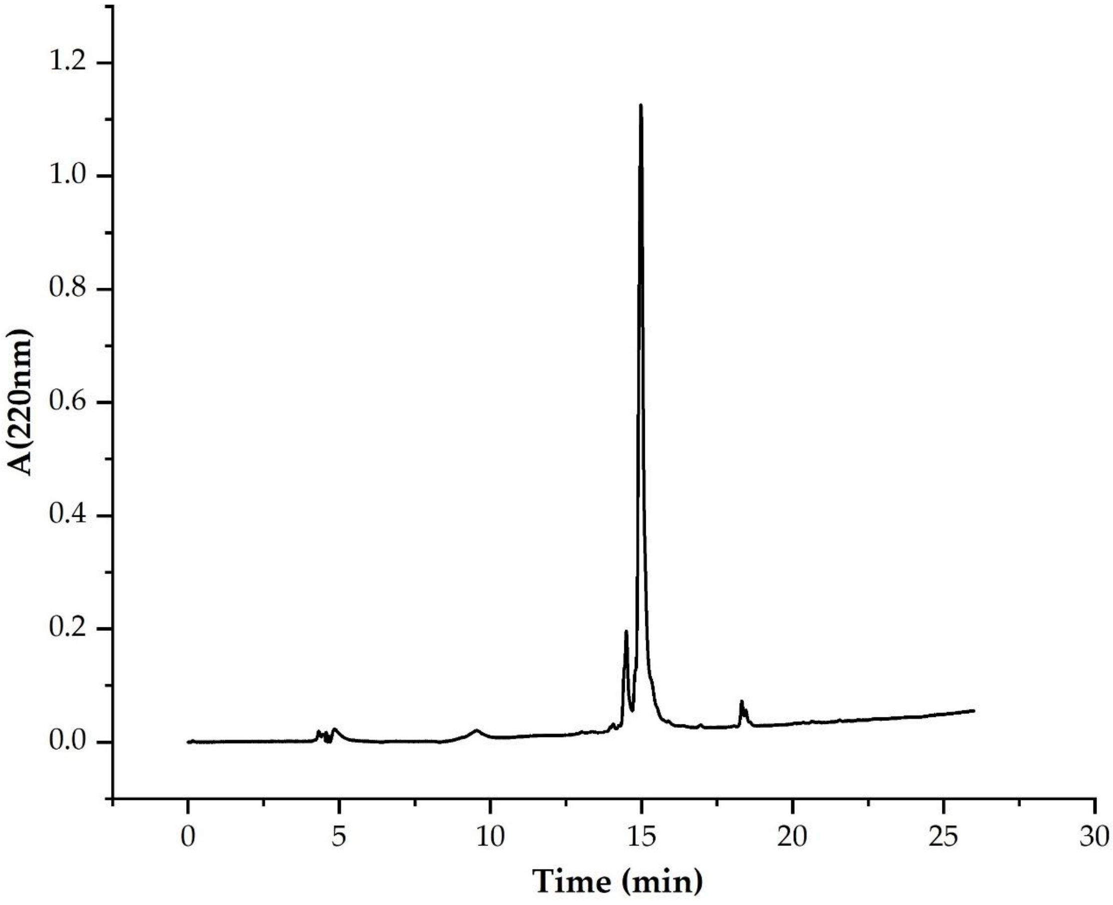

Figure S5.HPLC chromatogram of Dabcyl-azaGly-RRRRK(Cf). Retention time was obtained on Hypersil Hypurity C18 column (4.6 mm x 150 mm, 5 µm, 190 Å). The applied linear gradient elution was 0 min 0% B, 2 min 0% B, 22 min 90% B at 1 mL/min flow rate. The detection was carried out at λ = 220 nm. R_t = 15.0 min

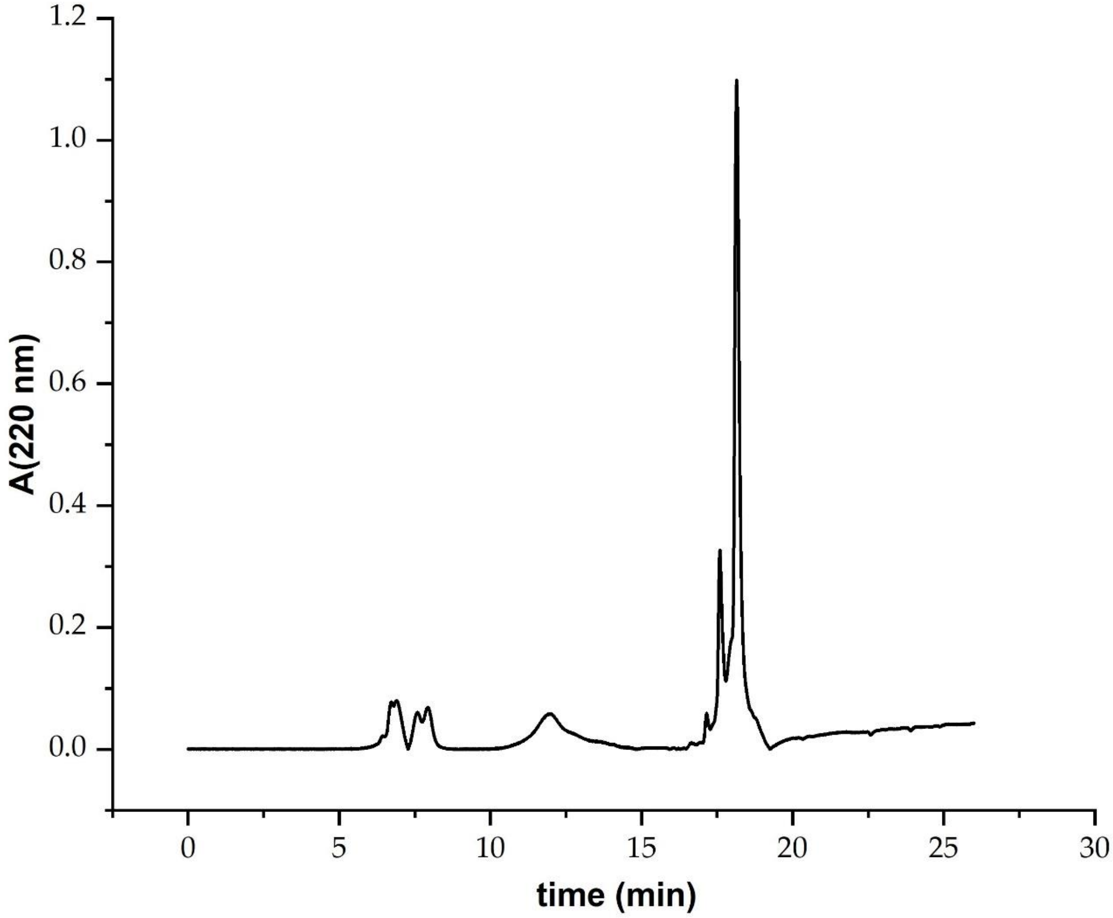

Figure S6.HPLC chromatogram of Dabcyl-RRWWRRK(Cf). Retention time was obtained on Hypersil Hypurity C18 column (4.6 mm x 150 mm, 5 µm, 190 Å). The applied linear gradient elution was 0 min 0% B, 2 min 0% B, 22 min 90% B at 1 mL/min flow rate. The detection was carried out at λ = 220 nm.

R_t = 18.2 min

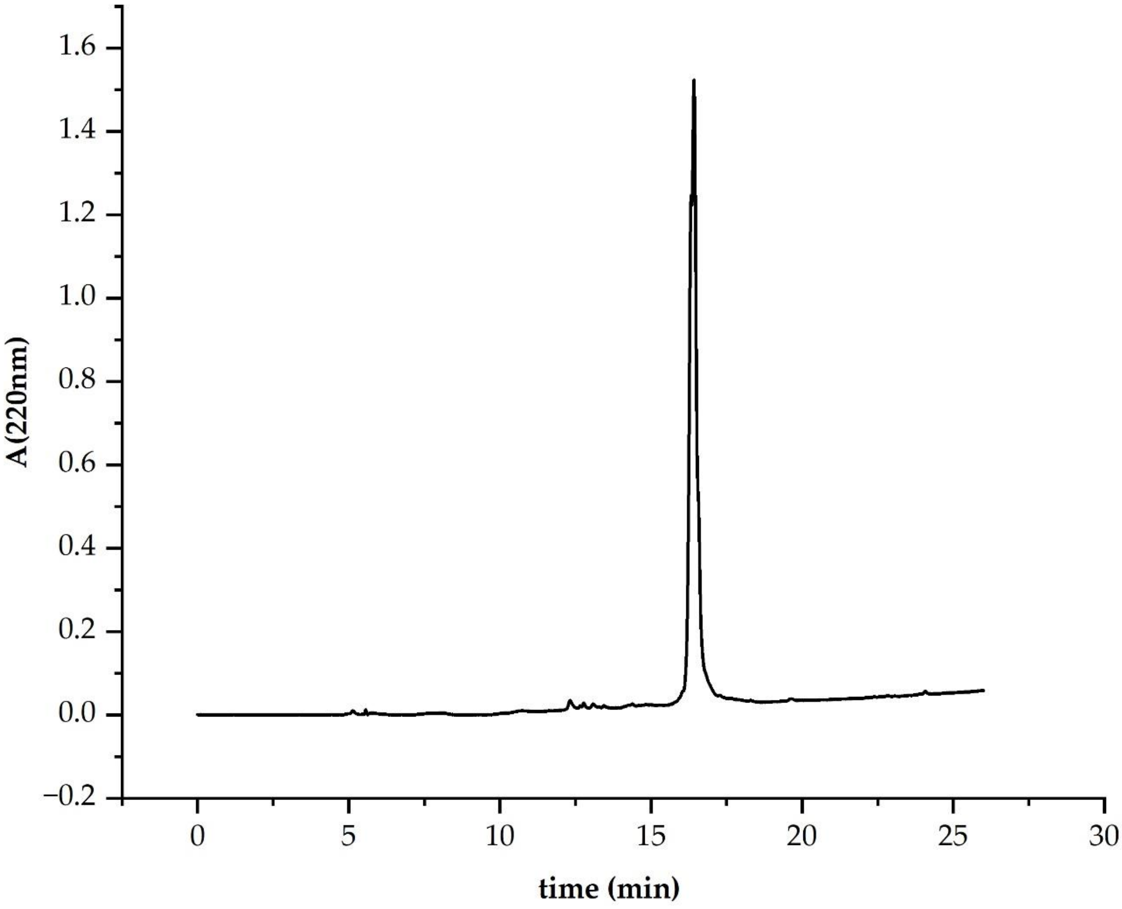

Figure S7. HPLC chromatogram of Dabcyl-RR-azaGly-azaGly-RRK(Cf). Retention time was obtained on Hypersil Hypurity C18 column (4.6 mm x 150 mm, 5 µm, 190 Å). The applied linear gradient elution was 0 min 0% B, 2 min 0% B, 22 min 90% B at 1 mL/min flow rate. The detection was carried out at λ = 220 nm. R_t = 16.4 min

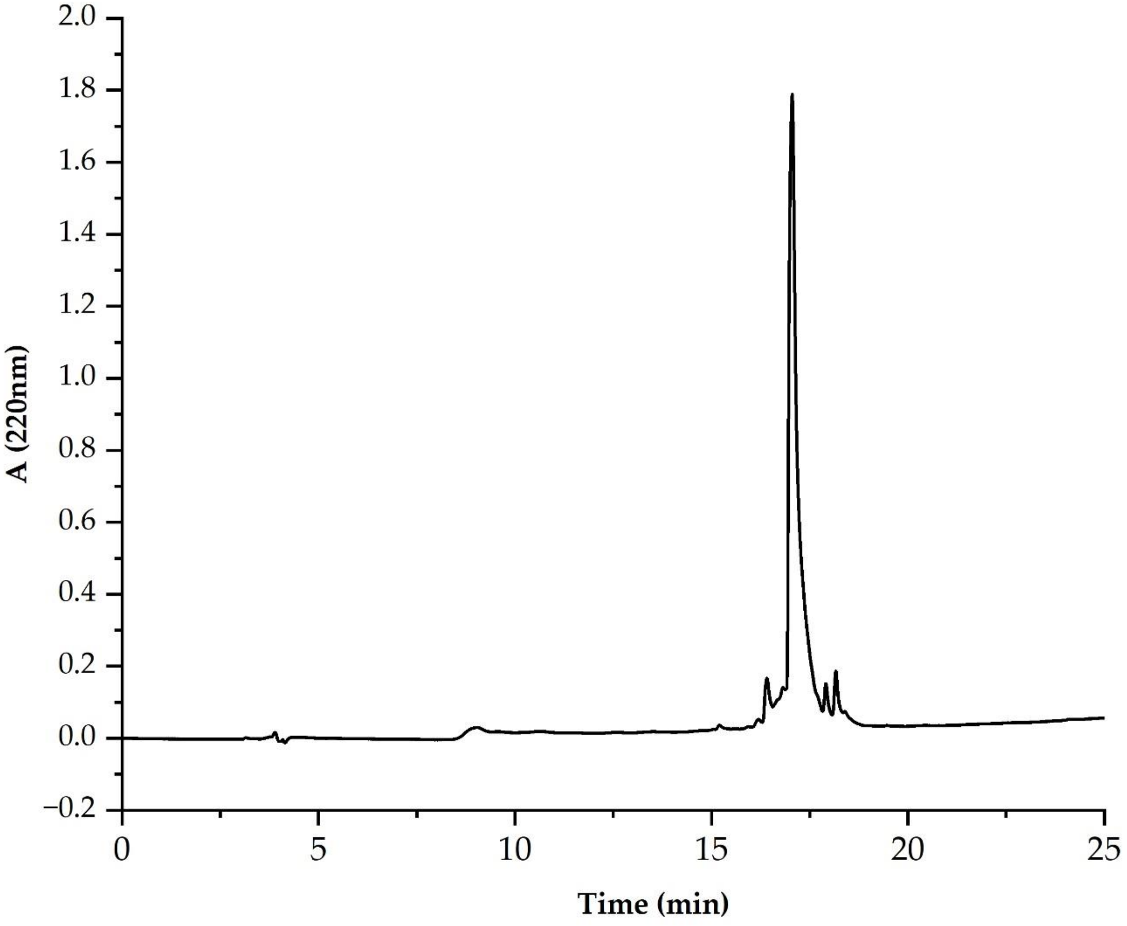

Figure S8. HPLC chromatogram of Dabcyl-RRWRRWK(Cf). Retention time was obtained on Hypersil Hypurity C18 column (4.6 mm x 150 mm, 5 µm, 190 Å). The applied linear gradient elution was 0 min 0% B, 2 min 0% B, 22 min 90% B at 1 mL/min flow rate. The detection was carried out at λ = 220 nm.

R_t = 17.1 min

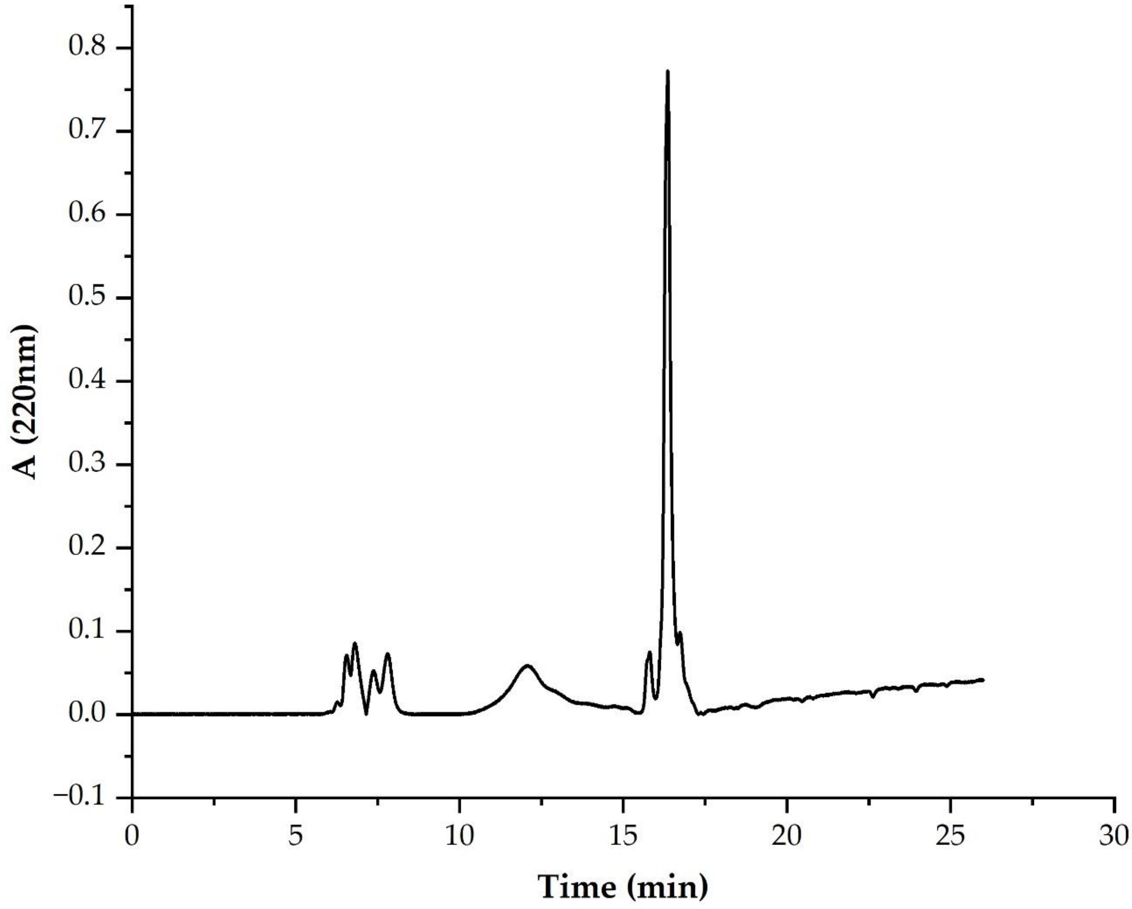

Figure S9. HPLC chromatogram of Dabcyl-RR-azaGly-RR-azaGly-K(Cf). Retention time was obtained on Hypersil Hypurity C18 column (4.6 mm x 150 mm, 5 μm, 190 Å). The applied linear gradient elution was 0 min 0% B, 2 min 0% B, 22 min 90% B at 1 mL/min flow rate. The detection was carried out at λ = 220 nm. R_t = 16.4 min

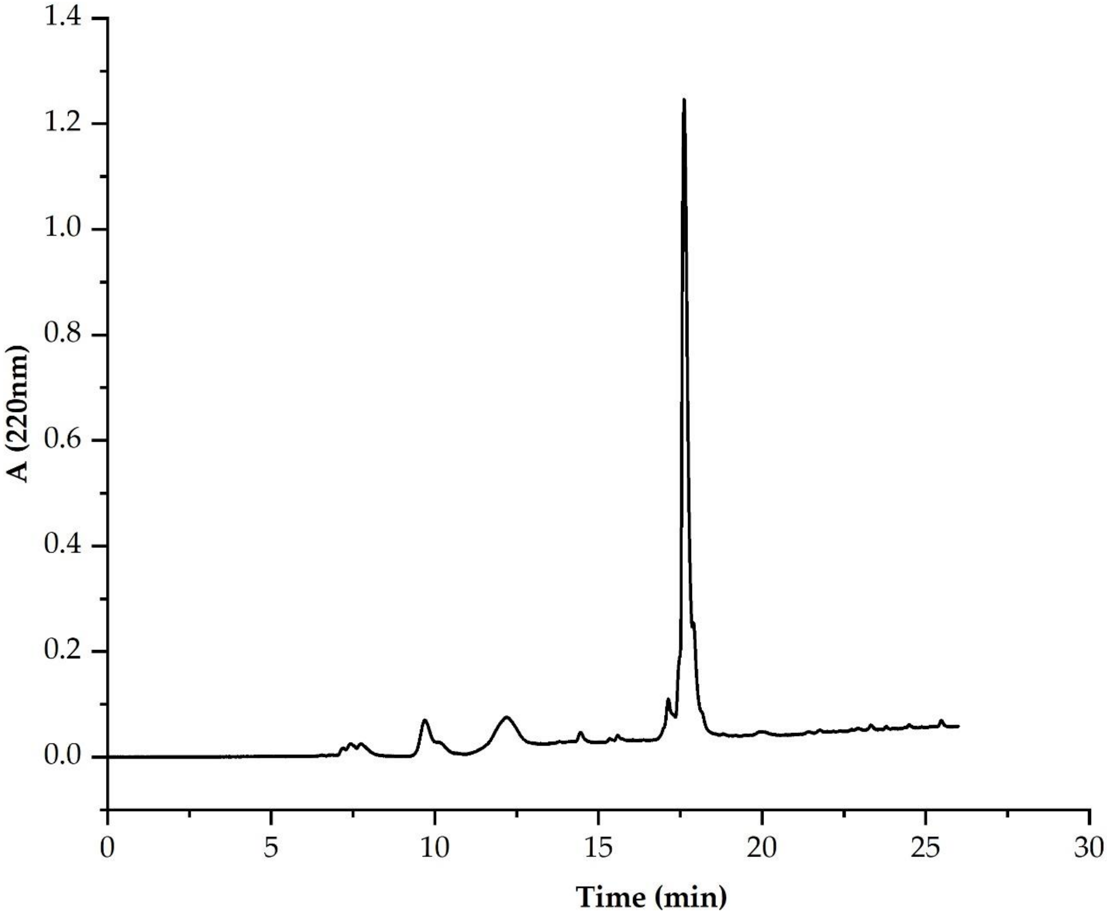

Figure S10. HPLC chromatogram of Dabcyl-WRRWRRK(Cf). Retention time was obtained on Hypersil Hypurity C18 column (4.6 mm x 150 mm, 5 μm, 190 Å). The applied linear gradient elution was 0 min 0% B, 2 min 0% B, 22 min 90% B at 1 mL/min flow rate. The detection was carried out at λ = 220 nm. R_t = 17.6 min

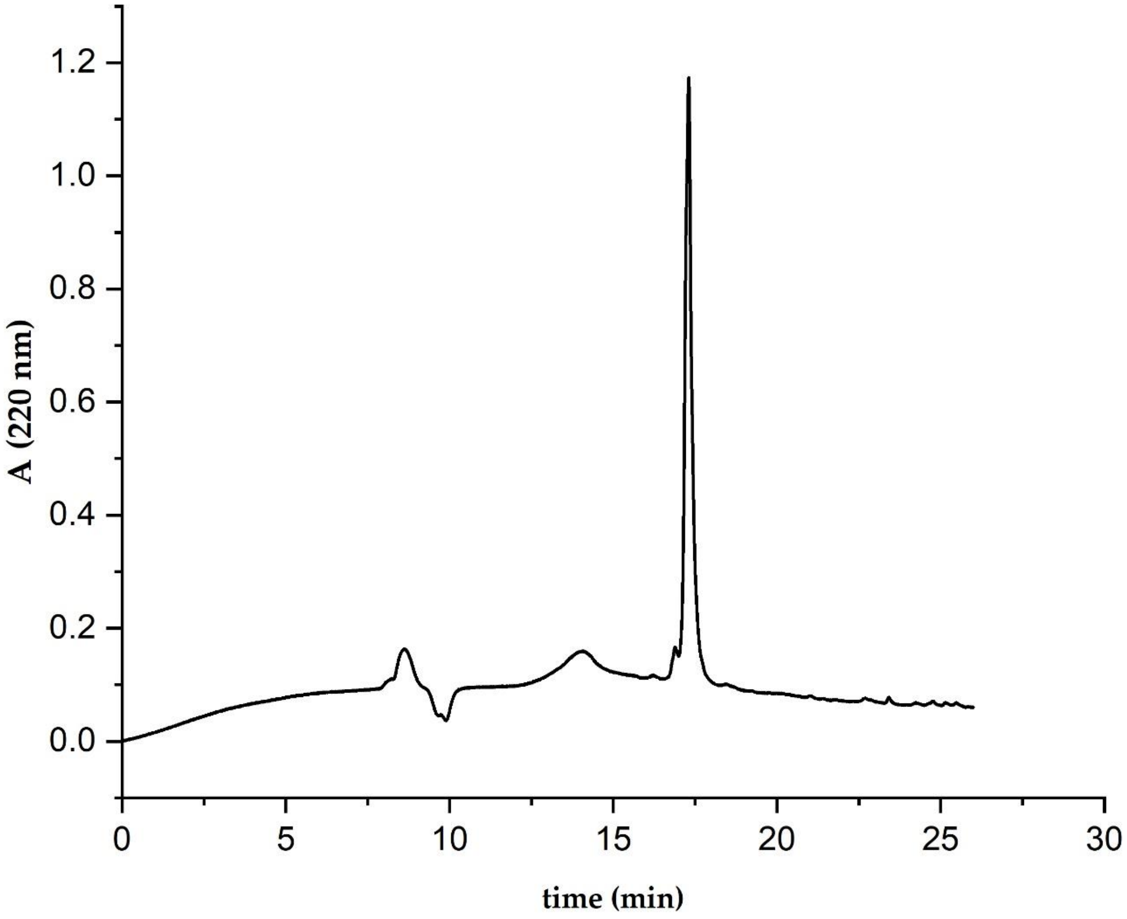

Figure S11. HPLC chromatogram of Dabcyl-azaGly-RR-azaGly-RRK(Cf). Retention time was obtained on Hypersil Hypurity C18 column (4.6 mm x 150 mm, 5 μm, 190 Å). The applied linear gradient elution was 0 min 0% B, 2 min 0% B, 22 min 90% B at 1 mL/min flow rate. The detection was carried out at λ = 220 nm. R_t = 17.3 min

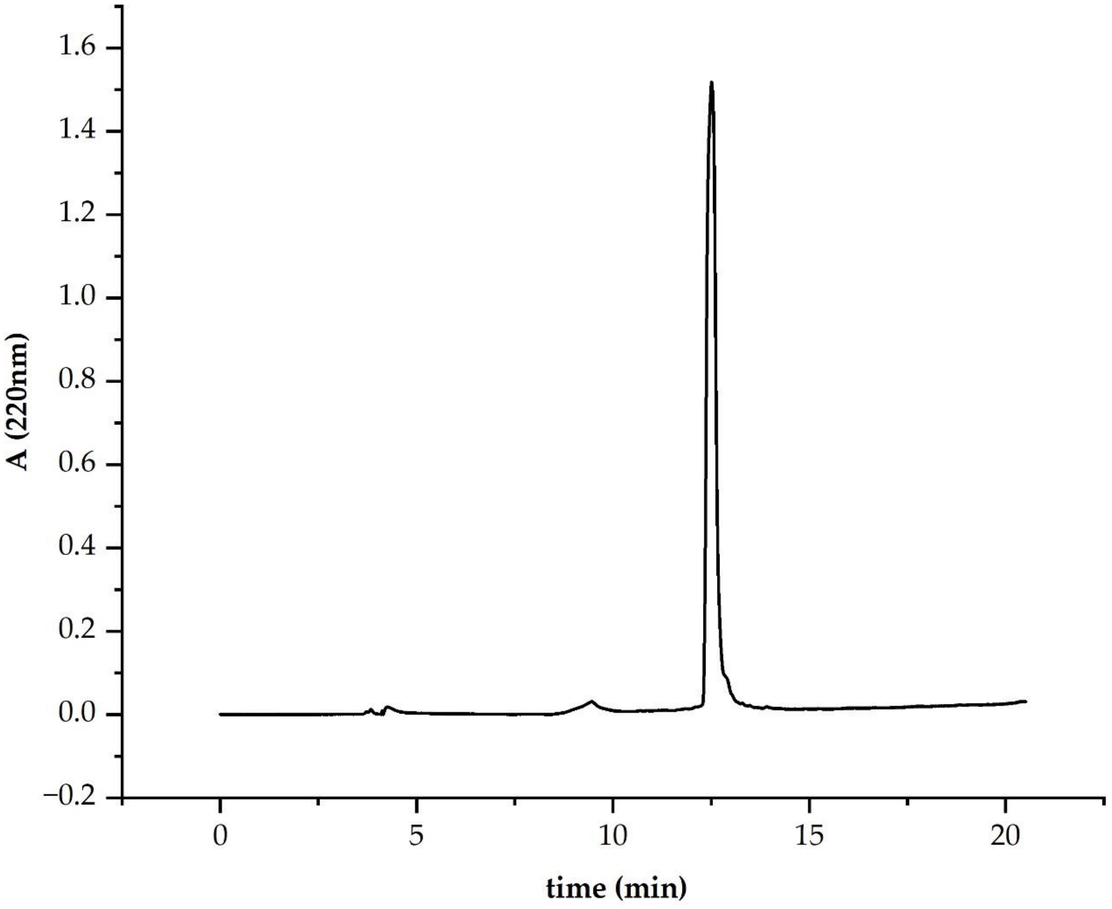

Figure S12. HPLC chromatogram of Cf-RRRRRRRR. Retention time was obtained on Hypersil Hypurity C18 column (4.6 mm x 150 mm, 5 μm, 190 Å). The applied linear gradient elution was 0 min 0% B, 2 min 0% B, 22 min 90% B at 1 mL/min flow rate. The detection was carried out at λ = 220 nm.

R_t = 12.5 min

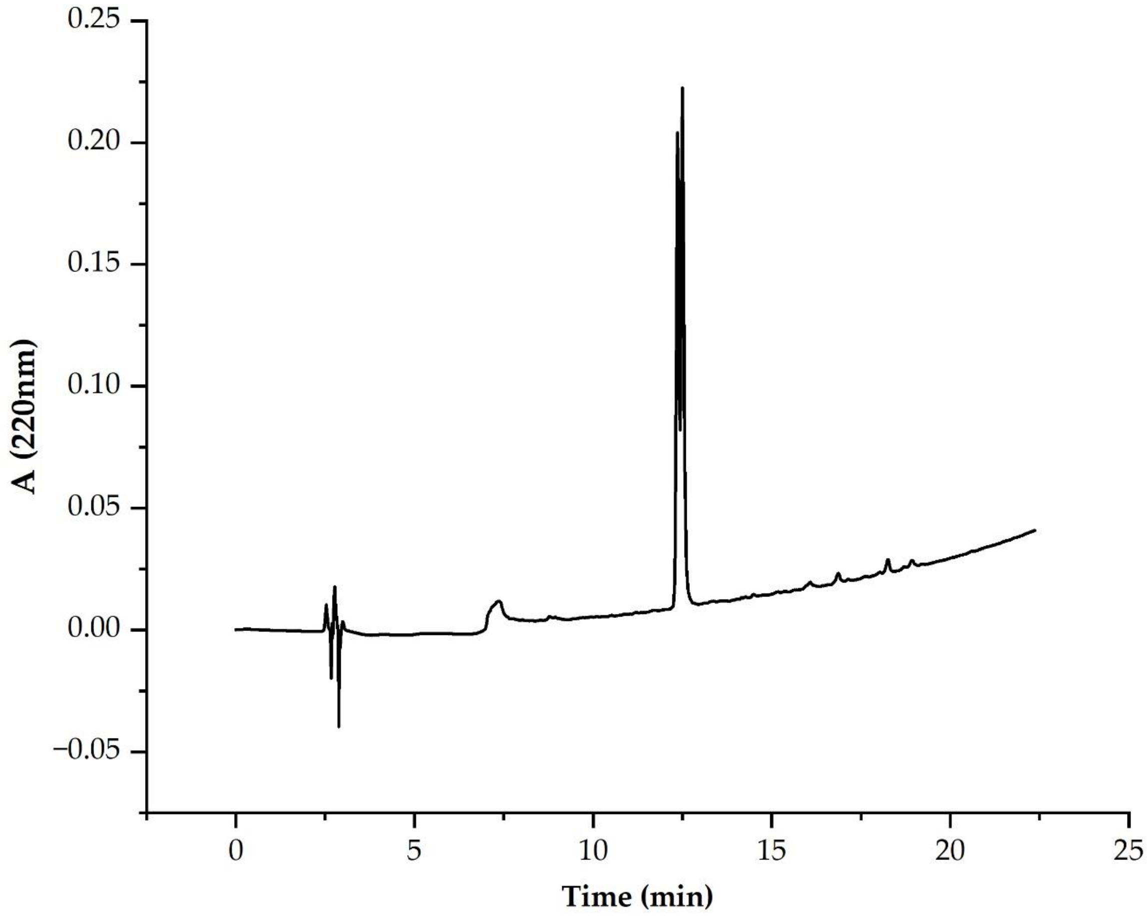

Figure S13. HPLC chromatogram of Dabcyl-RRRRK(Cf). Retention time was obtained on Hypersil Hypurity C18 column (4.6 mm x 150 mm, 5 µm, 190 Å). The applied linear gradient elution was 0 min 0% B, 2 min 0% B, 22 min 90% B at 1 mL/min flow rate. The detection was carried out at λ = 220 nm. R_t = 12.3 min

2.2 Mass spectrometry

The determination of the molecular weight of peptides and the fluorescently labelled peptides was performed by ESI-MS, using Bruker Amazon SL (Germany). All samples were dissolved in a solution containing water-acetonitrile solution (50:50) with 0.1% formic acid. Using a syringe pump, all samples were injected directly into the apparatus. The following parameters were implemented: capillary voltage: 4 kV, nebulizer gas: 10 psi, dry gas: 4 L/min, heated capillary temperature: 250 °C.

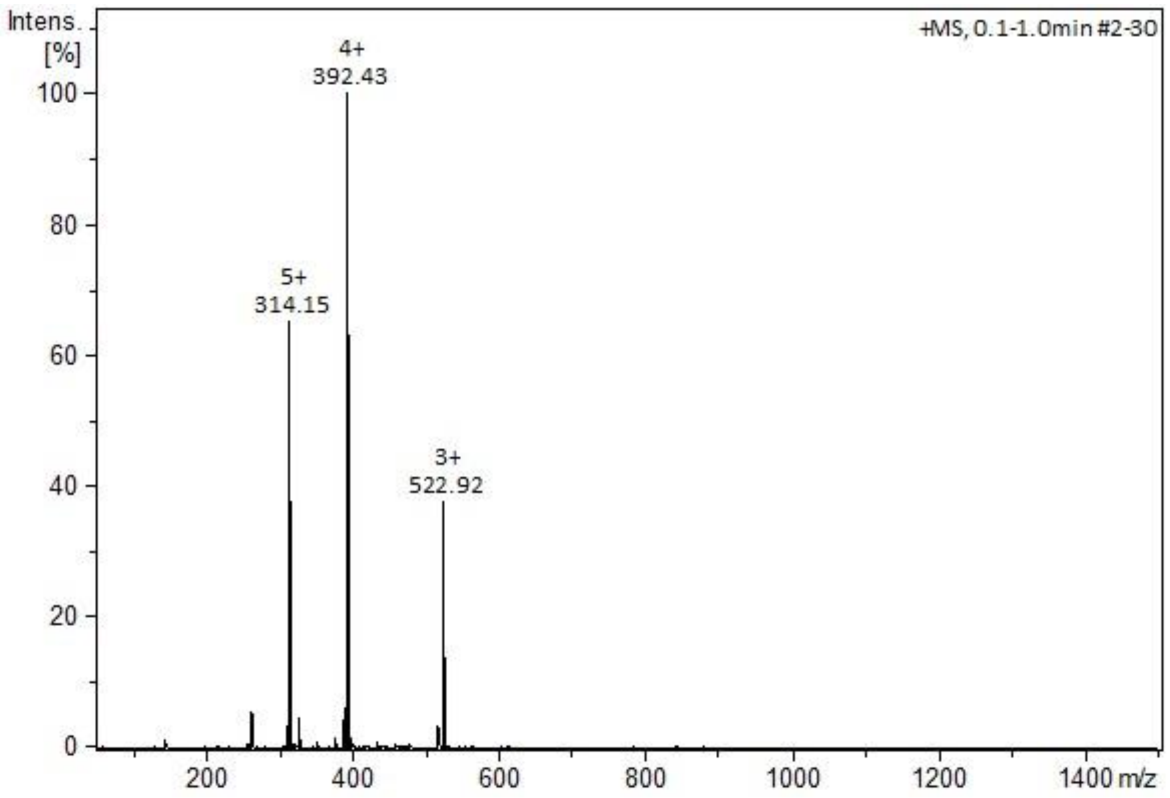

Figure S14. MS Spectrum of Dabcyl-RRWRRK(Cf). The i [illegible] he labelled peptide was determined using Bruker Amazon SL (Germany). The sample is dissolved in water-acetonitrile (50:50) with 0.1% formic acid. $M_{meas.}$= 1564.8 Da; $M_{cal.}$= 1565.2 Da

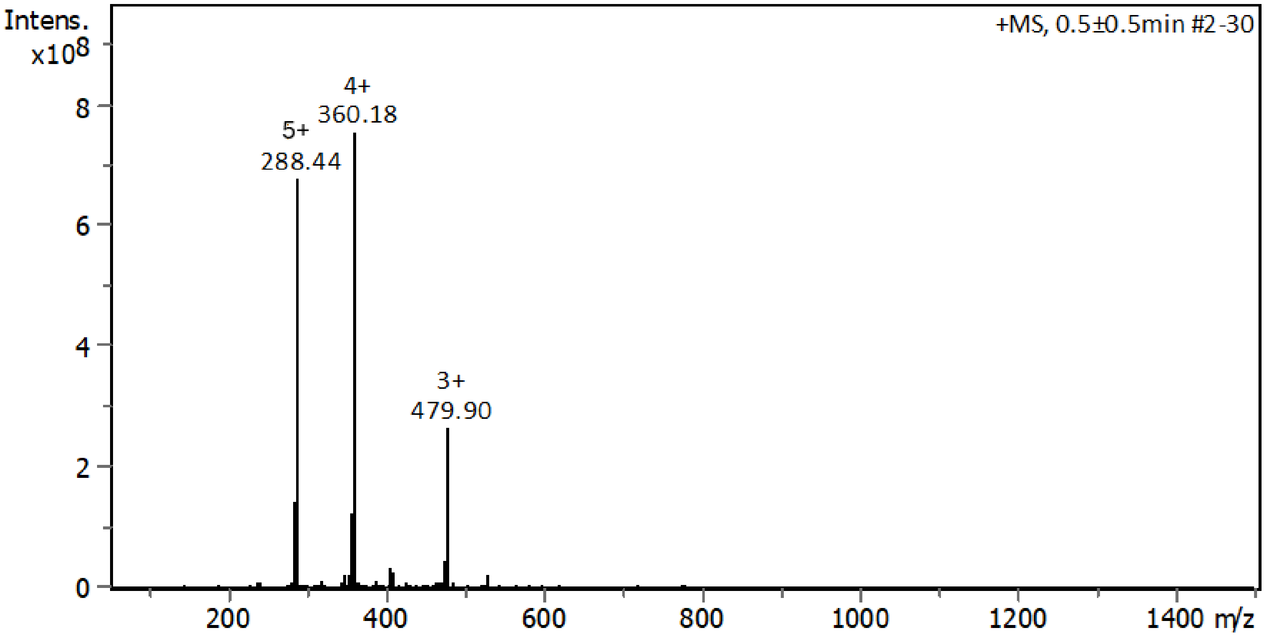

Figure S15. MS Spectrum of Dabcyl-RR-azaGly-RRK(Cf). The identification of the labelled peptide was determined using Bruker Amazon SL (Germany). The sample is dissolved in water-acetonitrile (50:50) with 0.1% formic acid. $M_{meas.}$= 1436.8 Da; $M_{cal.}$= 1436.7 Da

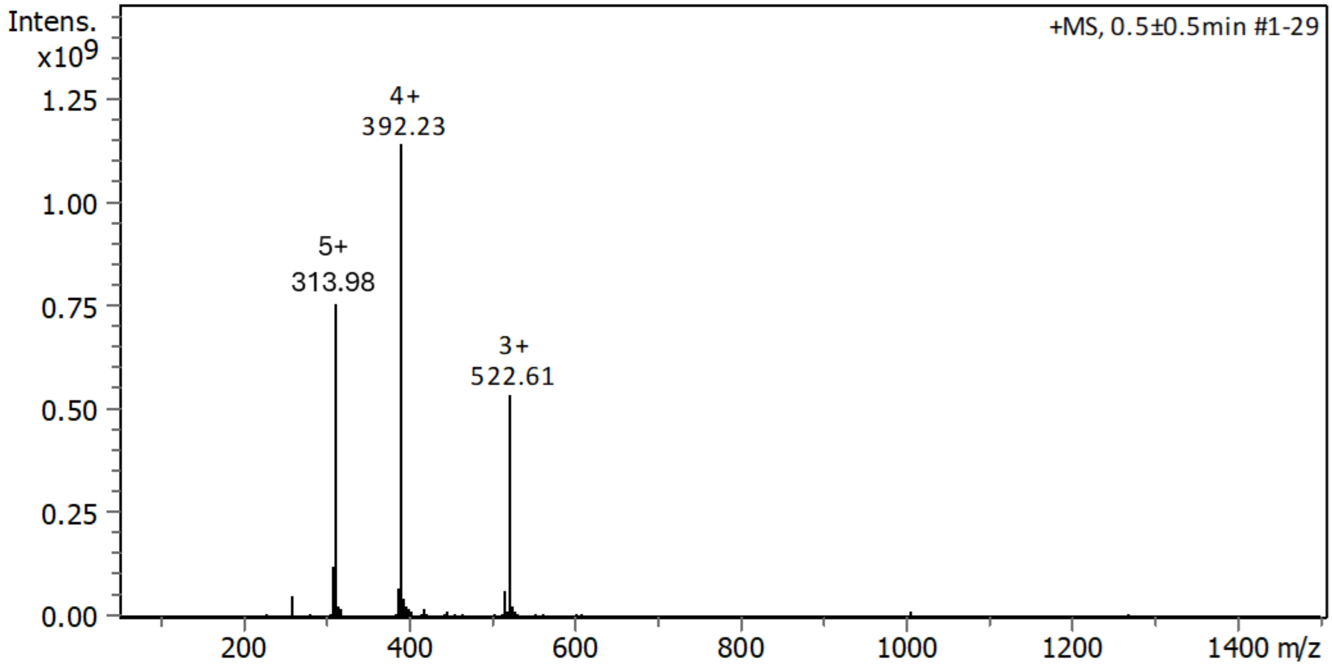

Figure S16. MS Spectrum of Dabcyl-RRRRWK(Cf). The identification of the labelled peptide was determined using Bruker Amazon SL (Germany). The sample is dissolved in water-acetonitrile (50:50) with 0.1% formic acid. $M_{meas.}$= 1565.7 Da; $M_{cal.}$= 1565.2 Da

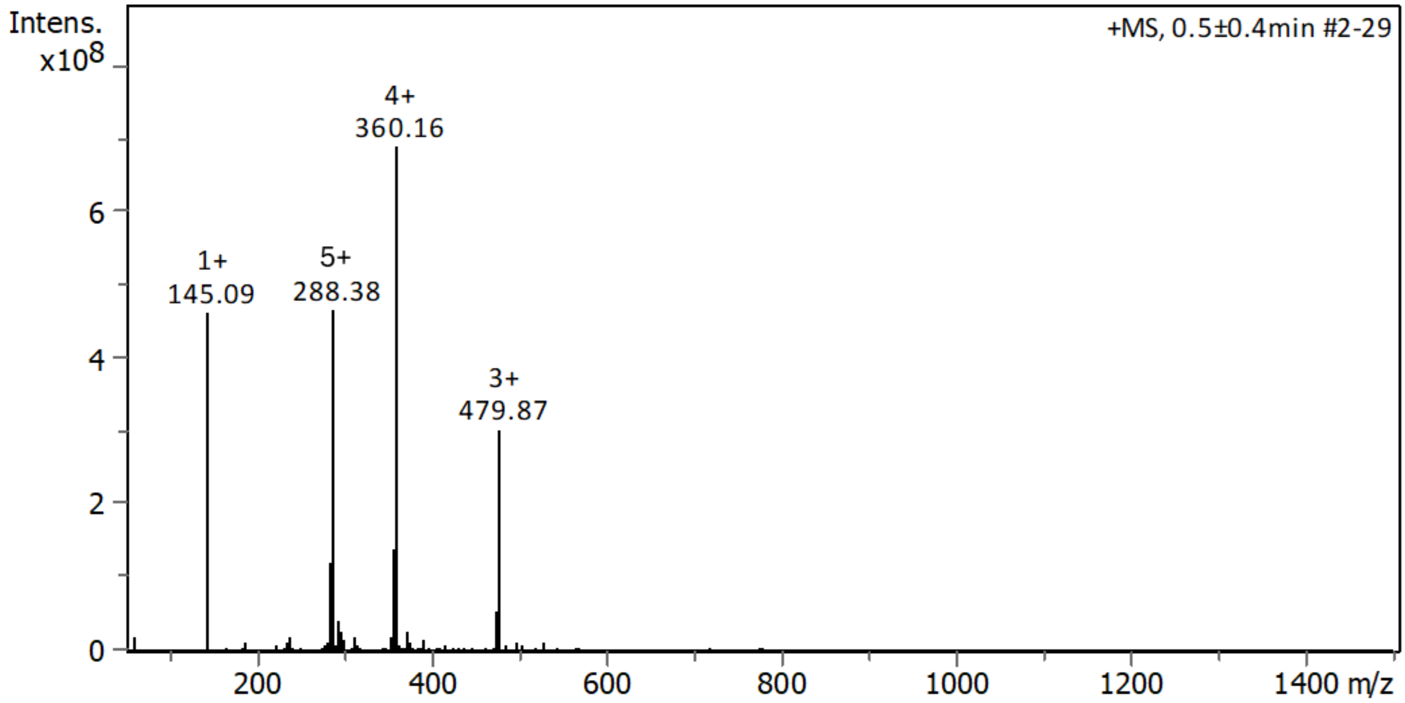

Figure S17. MS Spectrum of Dabcyl-RRRR-azaGly-K(Cf). The identification of the labelled peptide was determined using Bruker Amazon SL (Germany). The sample is dissolved in water-acetonitrile (50:50) with 0.1% formic acid. $M_{meas.}$= 1436.6 Da; $M_{cal.}$= 1436.7 Da

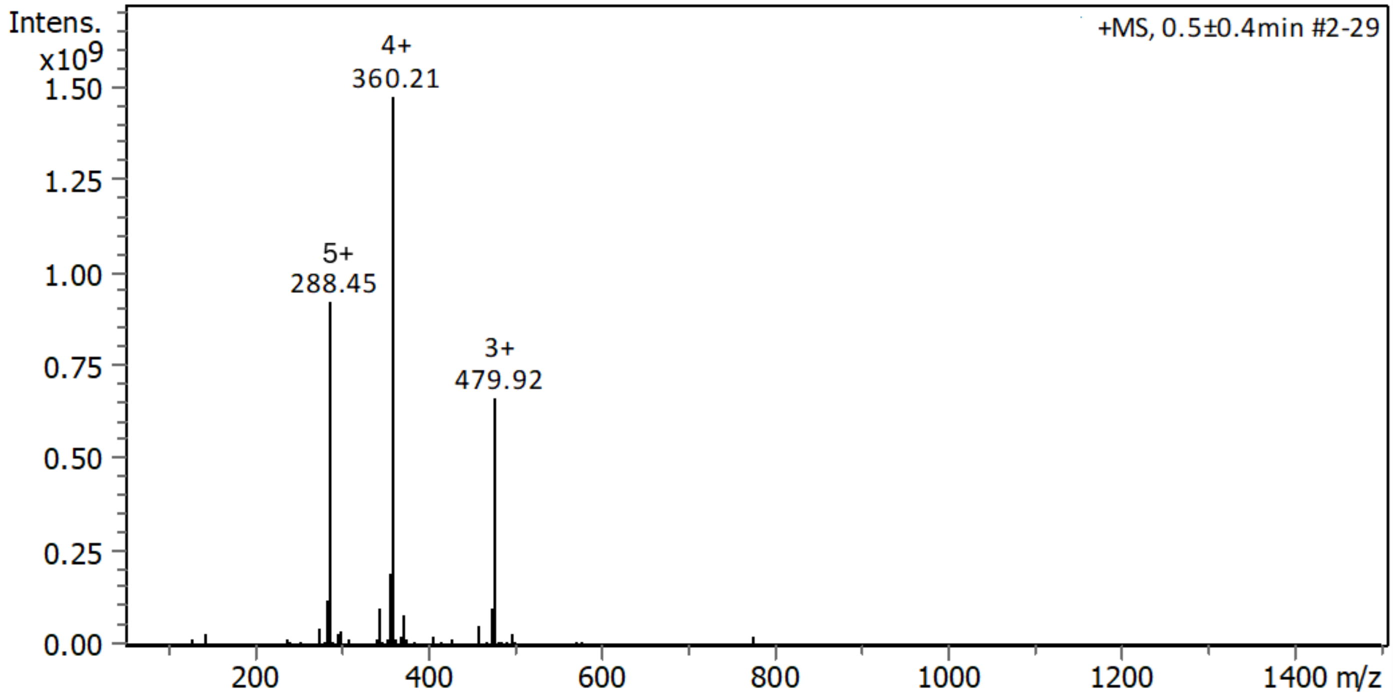

Figure S18. MS Spectrum of Dabcyl-azaGly-RRRRK(Cf).The identification of the labelled peptide was determined using Bruker Amazon SL (Germany). The sample is dissolved in water-acetonitrile (50:50) with 0.1% formic acid. $M_{meas.}$= 1436.7 Da; $M_{cal.}$= 1436.7 Da

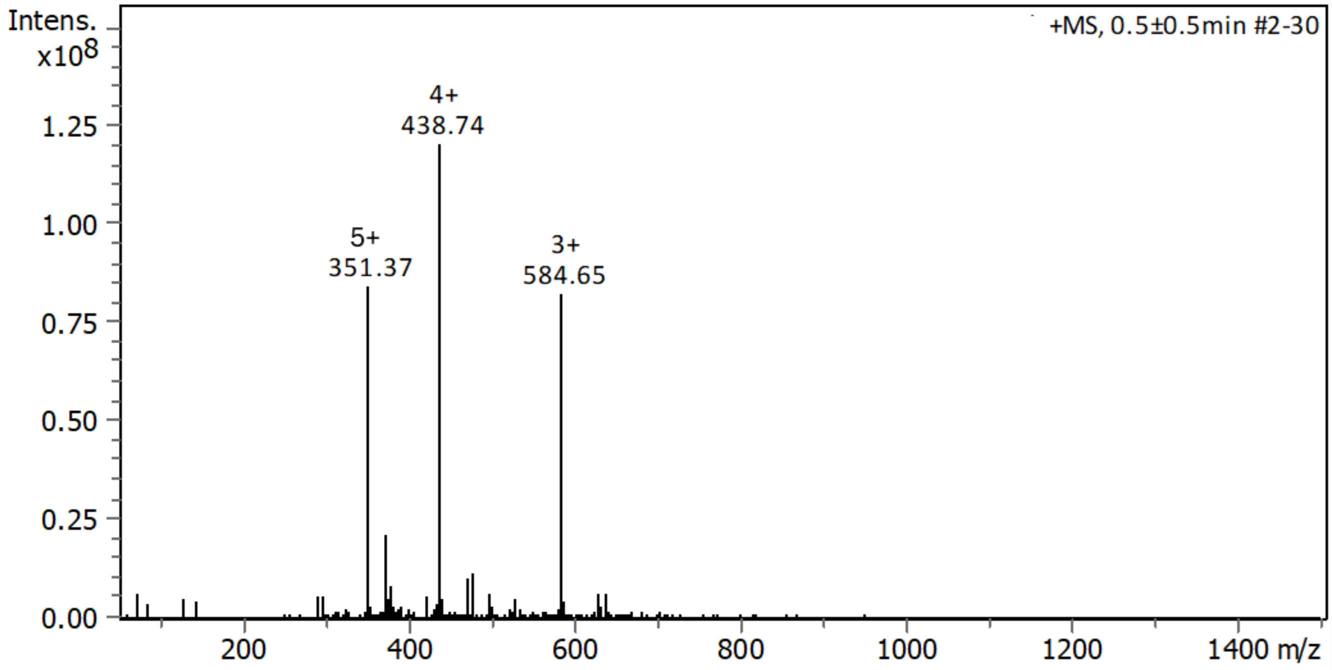

Figure S19. MS Spectrum of Dabcyl-RRWWRRK(Cf). The identification of the labelled peptide was determined using Bruker Amazon SL (Germany). The sample is dissolved in water-acetonitrile (50:50) with 0.1% formic acid. $M_{meas.}$= 1751.8 Da; $M_{cal.}$= 1751.8 Da

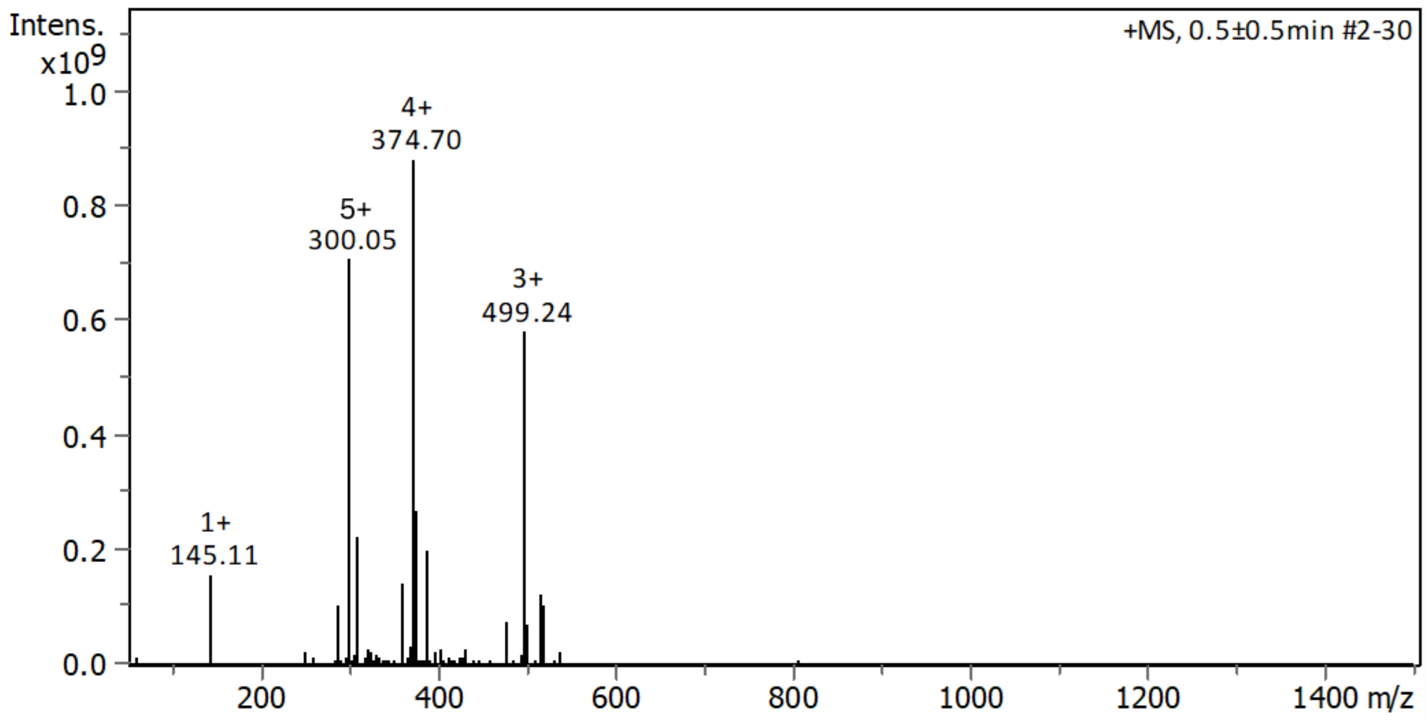

Figure S20. MS Spectrum of Dabcyl-RR-azaGly-azaGly-RRK(Cf).The identification of the labelled peptide was determined using Bruker Amazon SL (Germany). The sample is dissolved in water-acetonitrile (50:50) with 0.1% formic acid. $M_{meas.}$= 1494.6 Da; $M_{cal.}$= 1494.7 Da

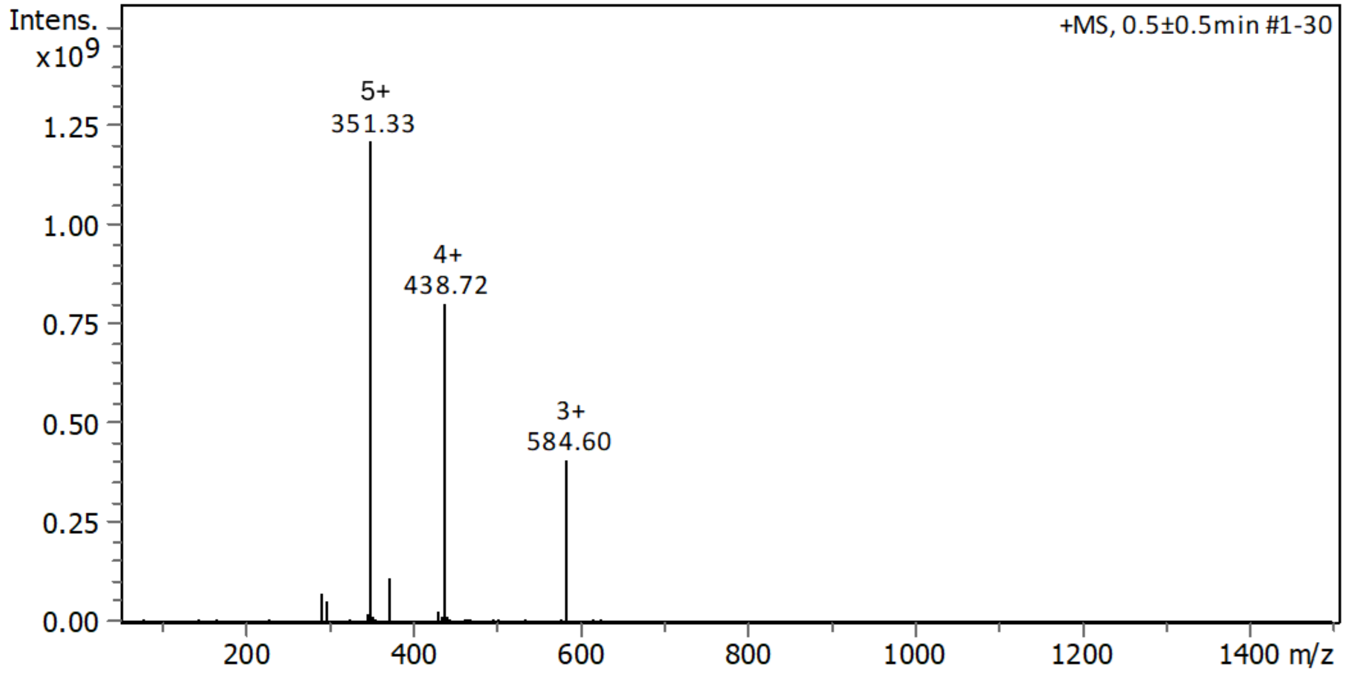

Figure S21. MS Spectrum of Dabcyl-RRWRRWKCf.The identification of the labelled peptide was determined using Bruker Amazon SL (Germany). The sample is dissolved in water-acetonitrile (50:50) with 0.1% formic acid. $M_{meas.}$= 1750.8 Da; $M_{cal.}$= 1750.8 Da

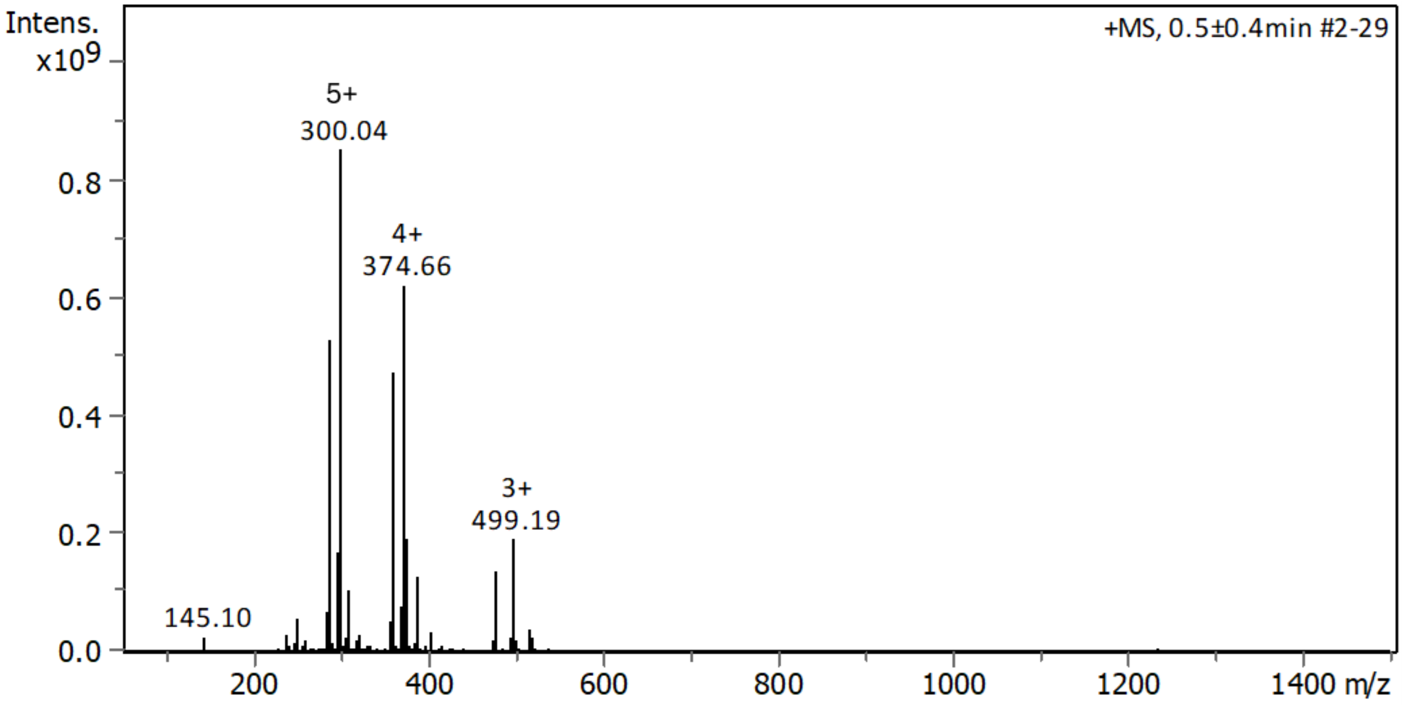

Figure S22. MS Spectrum of Dabcyl-RR-azaGly-RR-azaGlyK(Cf).The identification of the labelled peptide was determined using Bruker Amazon SL (Germany). The sample is dissolved in water-acetonitrile (50:50) with 0.1% formic acid. $M_{meas.}$= 1494,5 Da; $M_{cal.}$= 1494.7 Da

The additional peaks (288.44 (5+), 360.16 (4+), and 479.85 (3+)) come from the minor peptide impurity which has only one azaglycine moiety in its sequence.

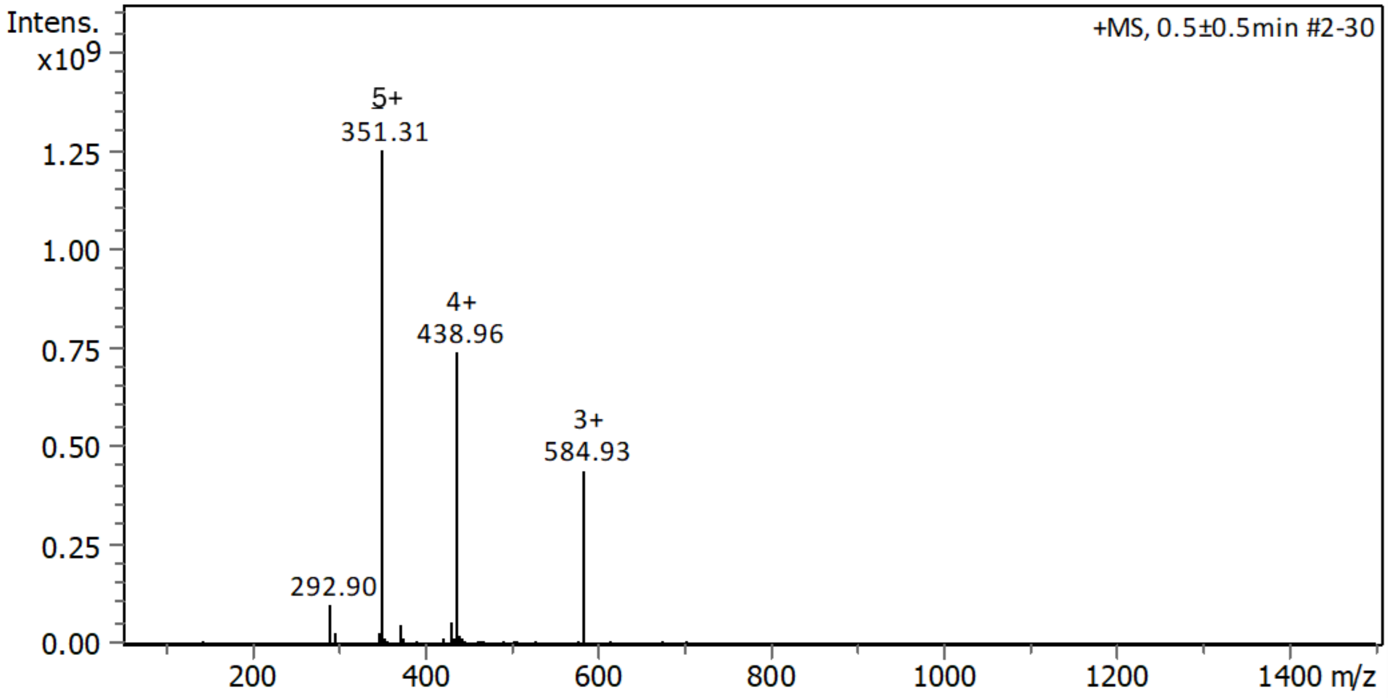

Figure S23. MS Spectrum of Dabcyl-WRRWRRK(Cf).The identification of the labelled peptide was determined using Bruker Amazon SL (Germany). The sample is dissolved in water-acetonitrile (50:50) with 0.1% formic acid. $M_{meas.}$= 1751.0 Da; $M_{cal.}$= 1750.8 Da

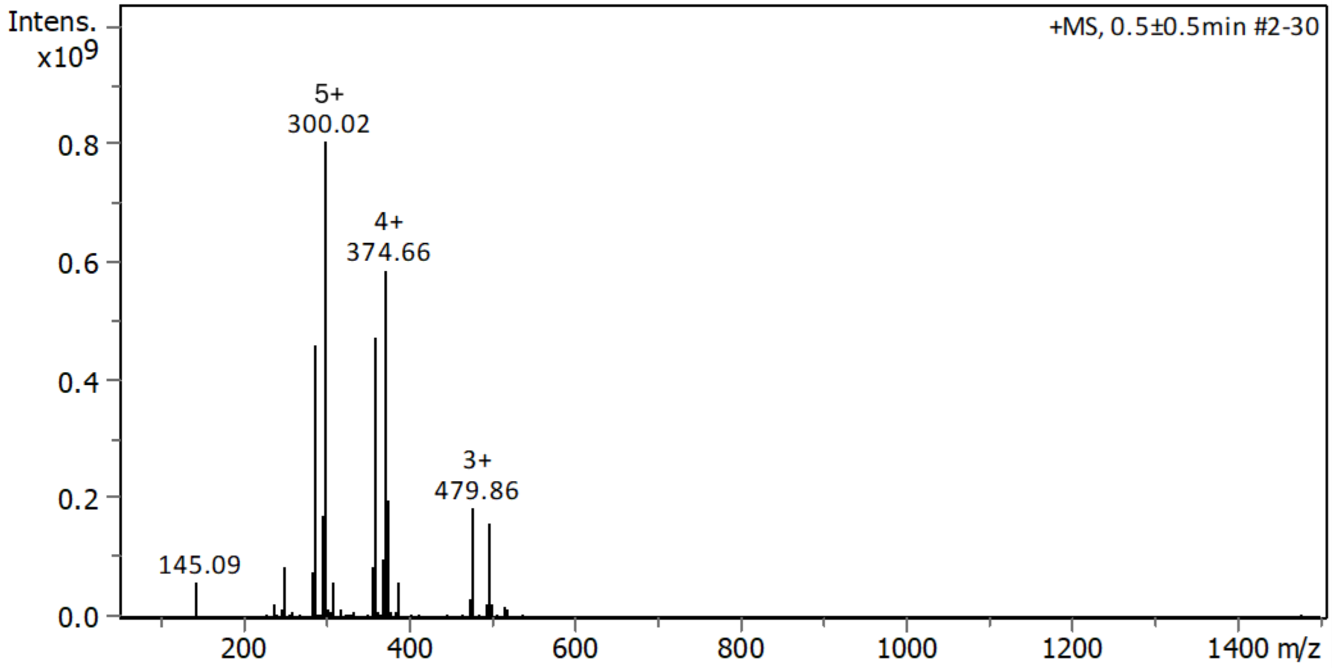

Figure S24. MS Spectrum of Dabcyl-azaGly-RR-azaGly-RRK(Cf). The identification of the labelled peptide was determined using Bruker Amazon SL (Germany). The sample is dissolved in water-acetonitrile (50:50) with 0.1% formic acid. $M_{meas.}$= 1494.7 Da; $M_{cal.}$= 1494.7 Da

The additional peaks (288.44 (5+), 360.16 (4+), and 479.86 (3+)) come from the minor peptide impurity which has only one azaglycine moiety in its sequence.

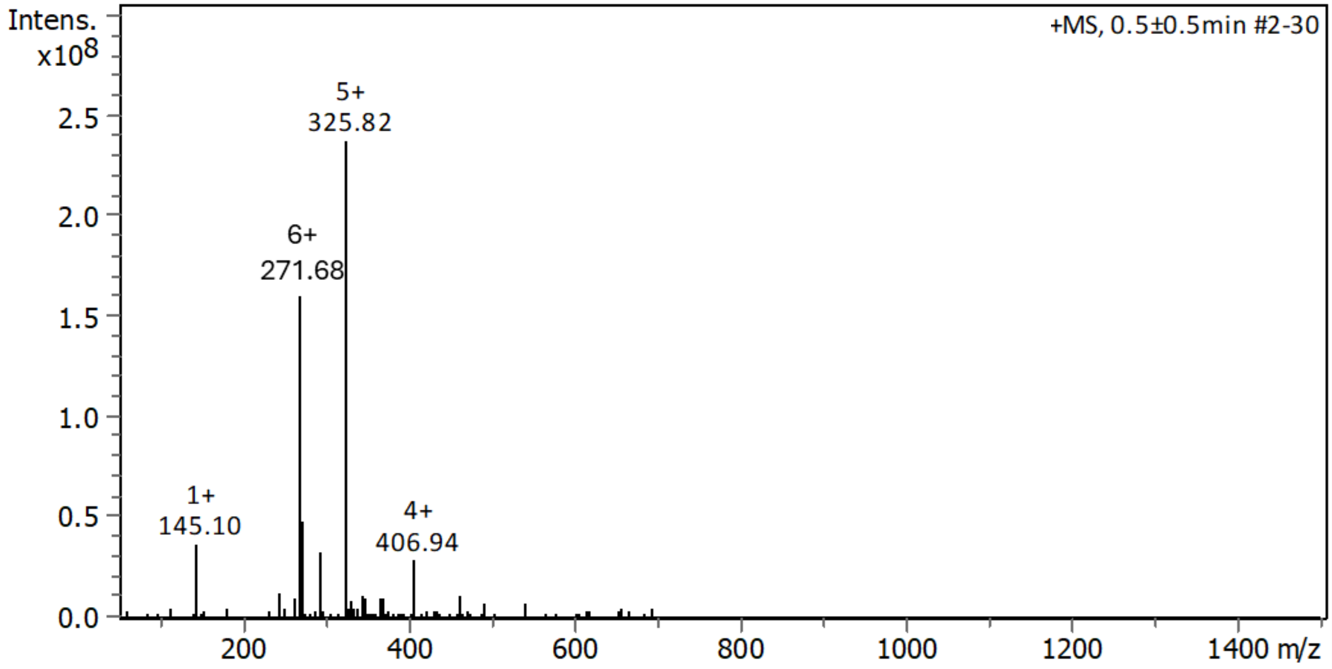

Figure S25. MS Spectrum of Cf-RRRRRRRR.The identification of the labelled peptide was determined using Bruker Amazon SL (Germany). The sample is dissolved in water-acetonitrile (50:50) with 0.1% formic acid. $M_{meas.}$= 1623.8 Da; $M_{cal.}$= 1623.9 Da

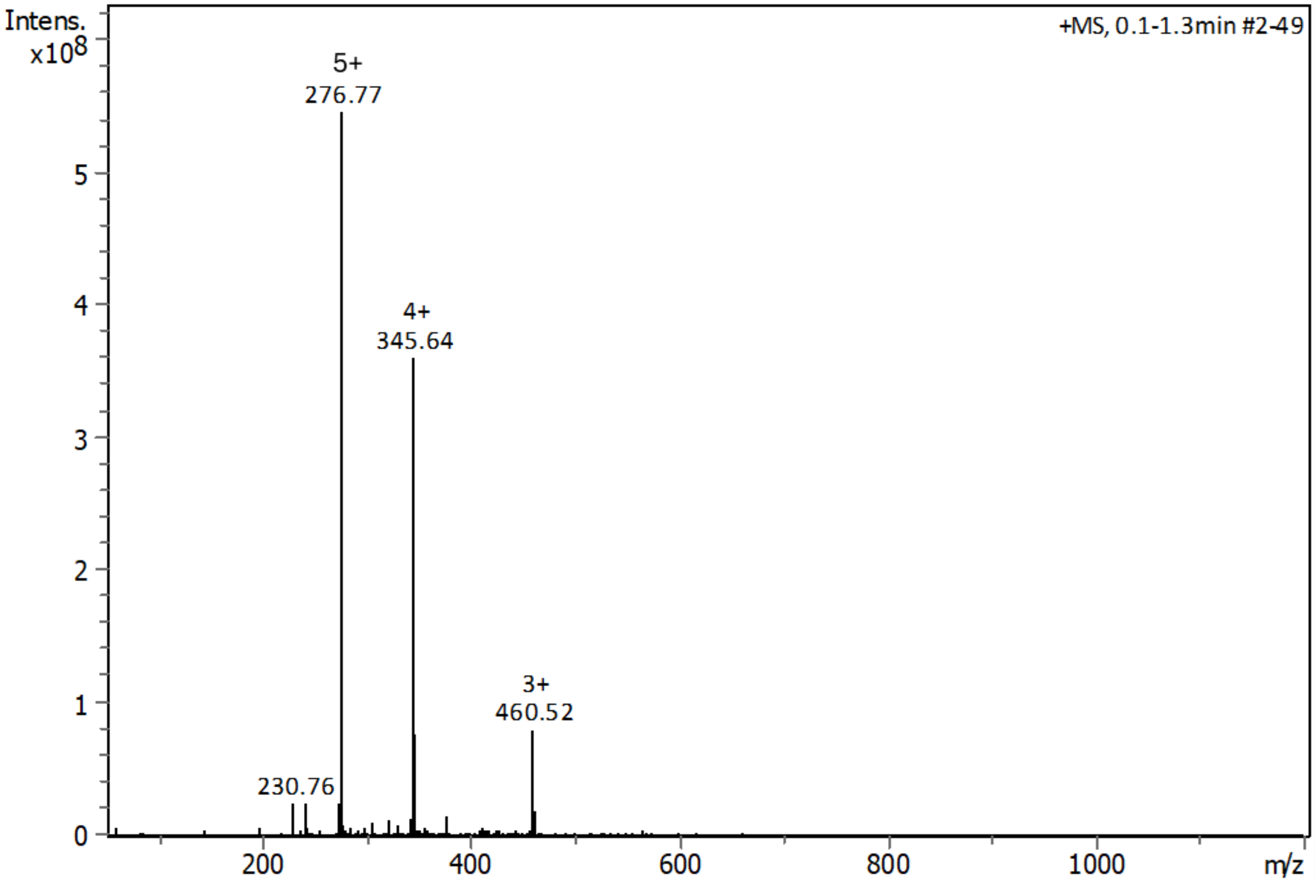

Figure S26. MS Spectrum of Dabcyl-RRRRK(Cf). The identification of the labelled peptide was determined using Bruker Amazon SL (Germany). The sample is dissolved in water-acetonitrile (50:50) with 0.1% formic acid. $M_{meas.}$= 1378.4Da; $M_{cal.}$= 1378.7Da

3. Cytotoxicity of Peptide Conjugates

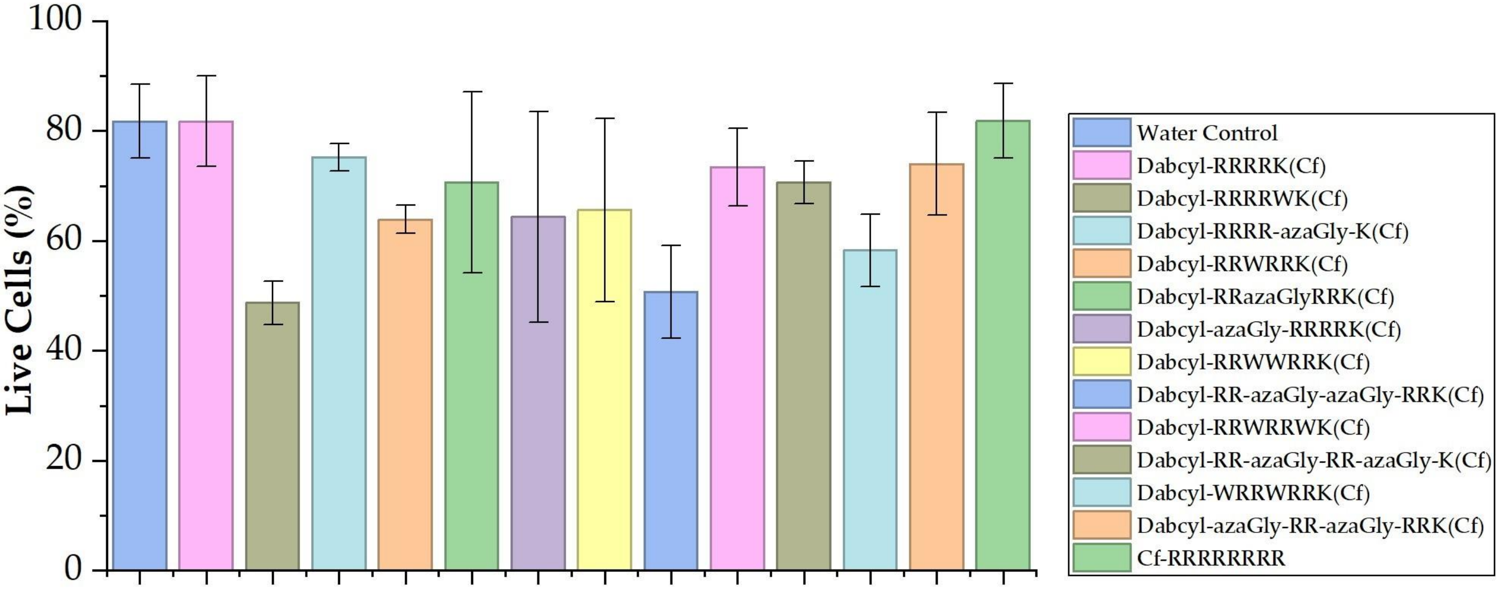

Figure S27. Evaluation of the cytotoxicity of peptides by examining the percentage of live cells by flow cytometry technique. Cells were incubated with 5 μM peptide solution for 90 min at 37°C.

4. Computational Calculations of Peptide Conformation and Membrane Interaction

Figure S28. SMD/LC-ωPBE-D3/3-21+G*-optimized structure of model peptides **1**-**9**.

**Model Peptides**	ϕ_1	ψ_1	ϕ_2	ψ_2	ϕ_3	ψ_3	ϕ_4	ψ_4	ϕ_5	ψ_5	ϕ_6	ψ_6		Polarizability (α)
Ac-**W**RRRRK-NHMe (**1**)	-61	-33	-59	-45	-68	-44	-58	-42	-74	-51	-99	-19		851.9
Ac-**G**RRRRK-NHMe (**2**)	-62	-46	-74	-28	-61	-46	-64	-46	-56	-51	-92	-23		703.4
Ac-**aG**RRRRK-NHMe*(**3**)	-71	-25	-63	-43	-64	-41	-59	-45	-65	-44	-76	-25		723.1
Ac-RR**W**RRK-NHMe (**4**)	-60	-35	-55	-44	-74	-46	-67	-35	-62	-41	-73	-42		847.8
Ac-RR**G**RRK-NHMe (**5**)	-52	-42	-58	-26	-62	-31	-99	-34	-75	-38	-120	14		709.2
Ac-RR**aG**RRK-NHMe (**6**)	-60	-34	-52	-49	-79	-29	-53	-48	-75	-43	-62	-41		699.8
Ac-RRRR**W**K-NHMe (**7**)	-56	-42	-57	-48	-62	-46	-63	-51	-86	-41	-71	-28		850.7
Ac-RRRR**G**K-NHMe (**8**)	-69	-22	-59	-51	-62	-40	-56	-48	-67	-41	-63	-39		701.5
Ac-RRRR**aG**K-NHMe (**9**)	-68	-23	-59	-51	-63	-40	-54	-47	-76	-29	-66	-38		697.5

*SMD/LC-ωPBE-D3/6-31+G(d) level of theory.

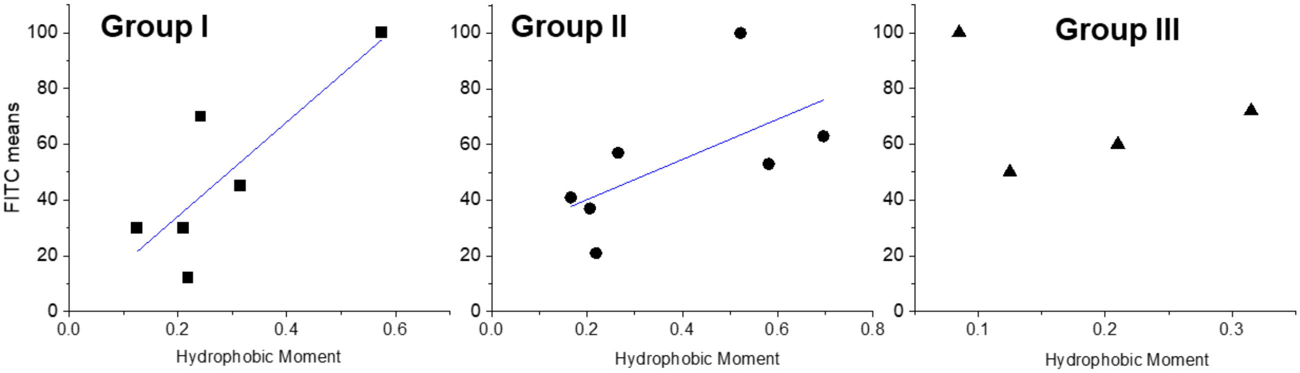

Figure S29. Hydrophobic moment vs FITC means model peptides (see Table 2).

5. Cellular uptake of peptides – histograms and dot plots

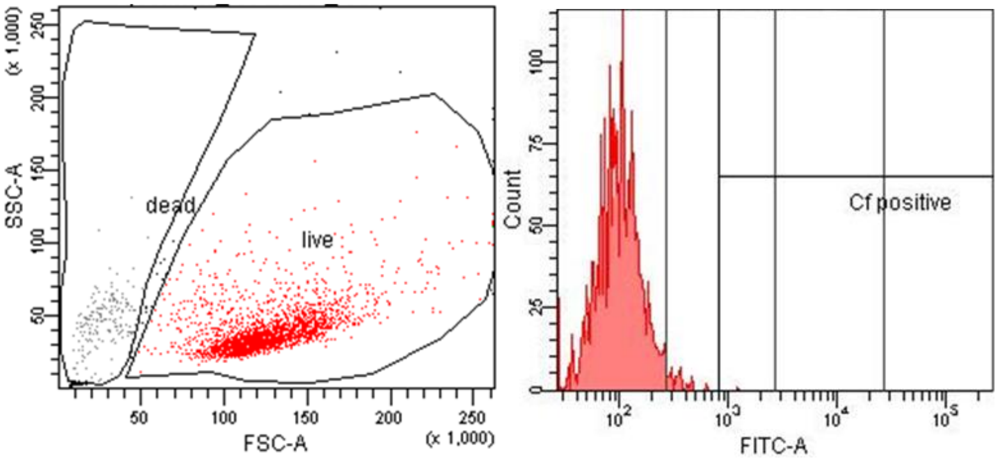

control

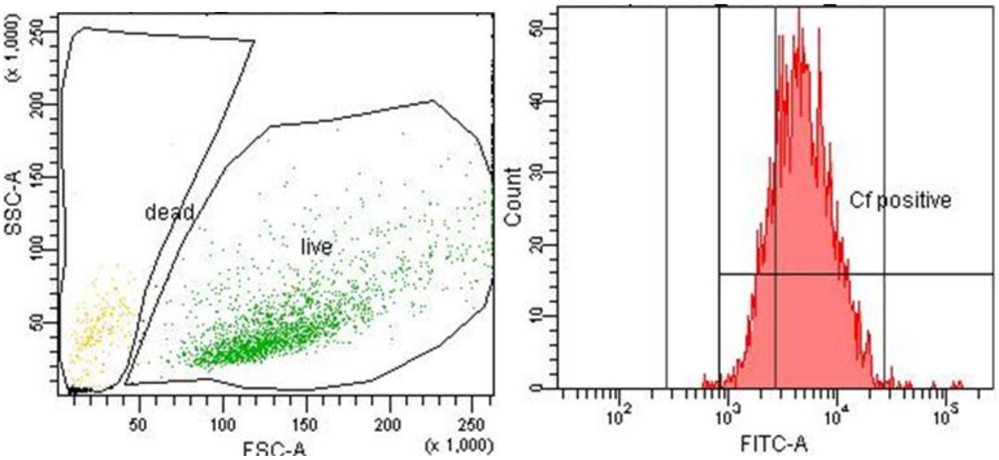

Dabcyl-RRWRRK(Cf)

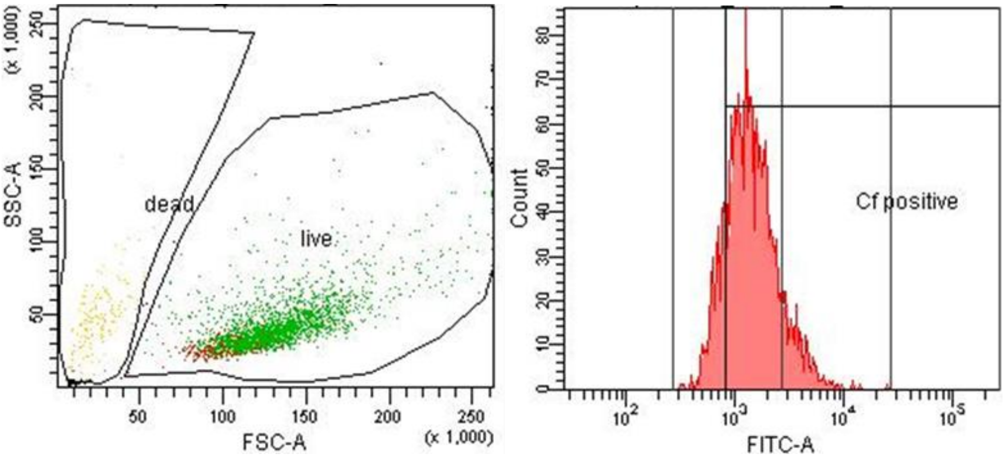

Dabcyl-RR-azaGly-RRK(Cf)

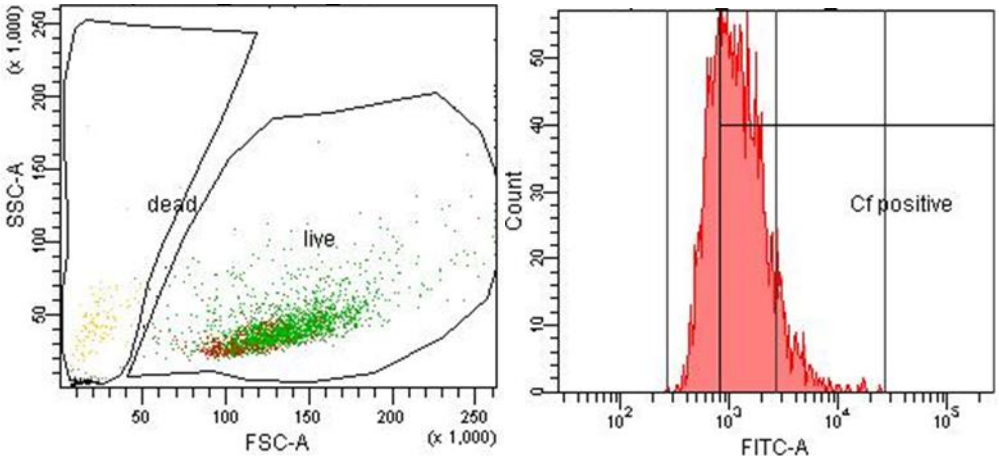

Dabcyl-azaGly-RRRRK(Cf)

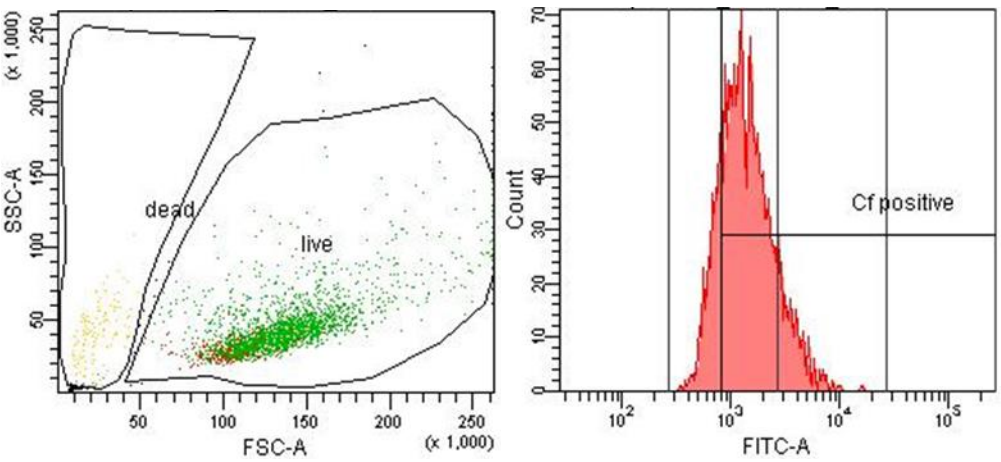

Dabcyl-azaGly-RR-azaGly-RRK(Cf)

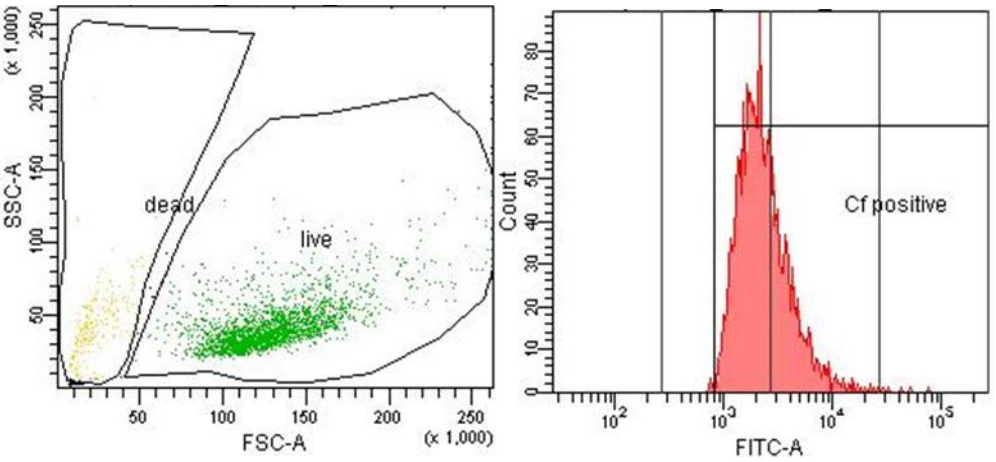

Dabcyl-RRWWRRRK(Cf)

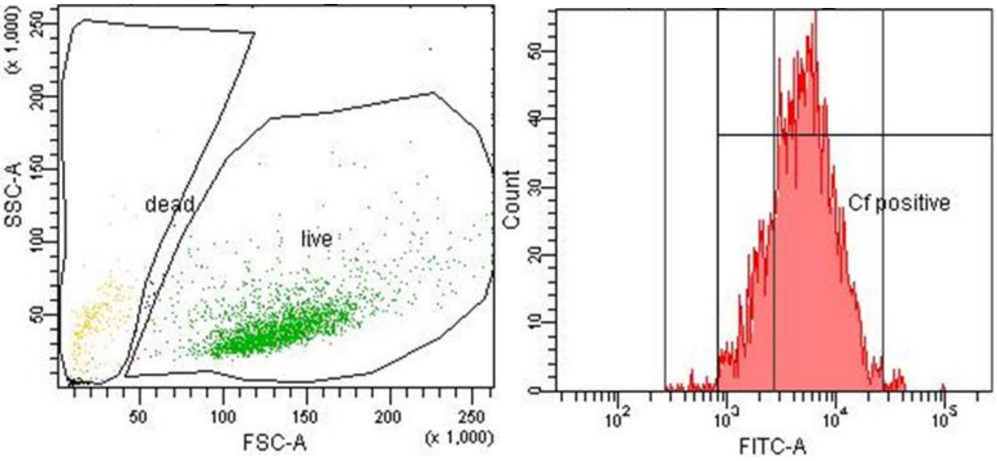

Cf-RRRRRRRR

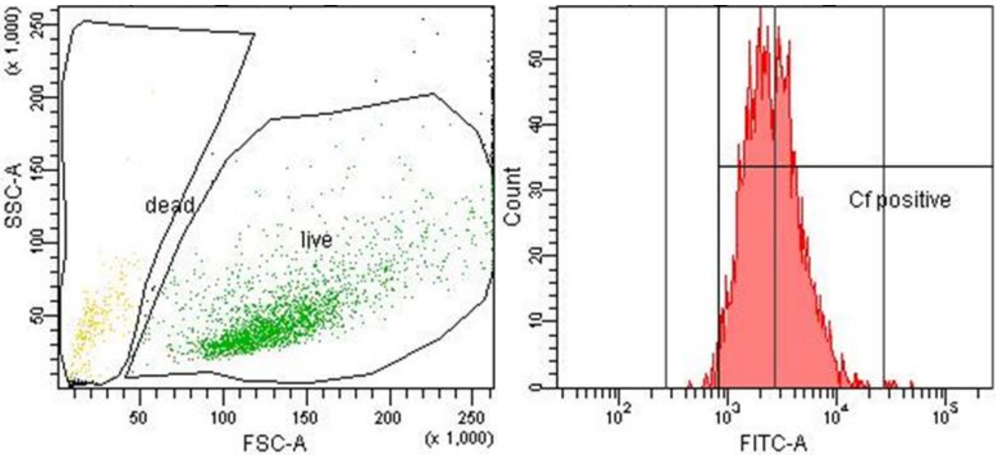

Dabcyl-RRWRRWK(Cf)

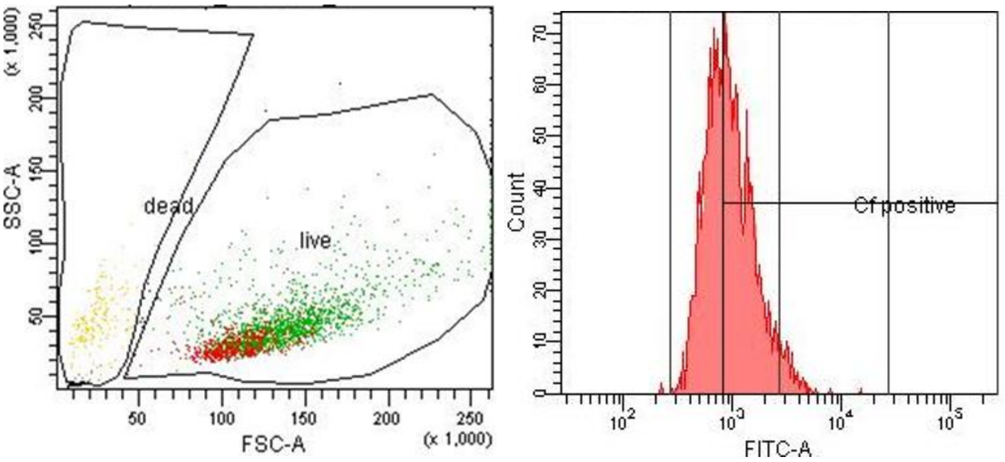

Dabcyl-RR-azaGly-RR-azaGly-K(Cf)

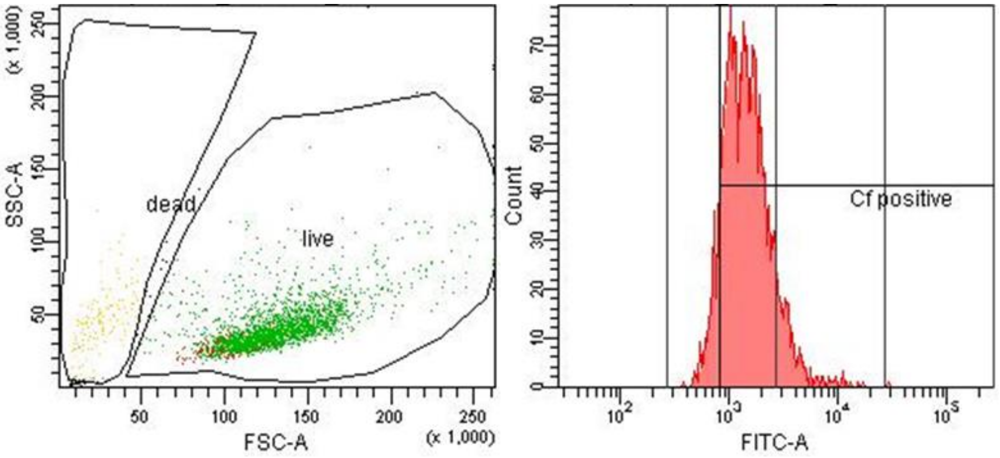

Dabcyl-RRRR-azaGly-K(Cf)

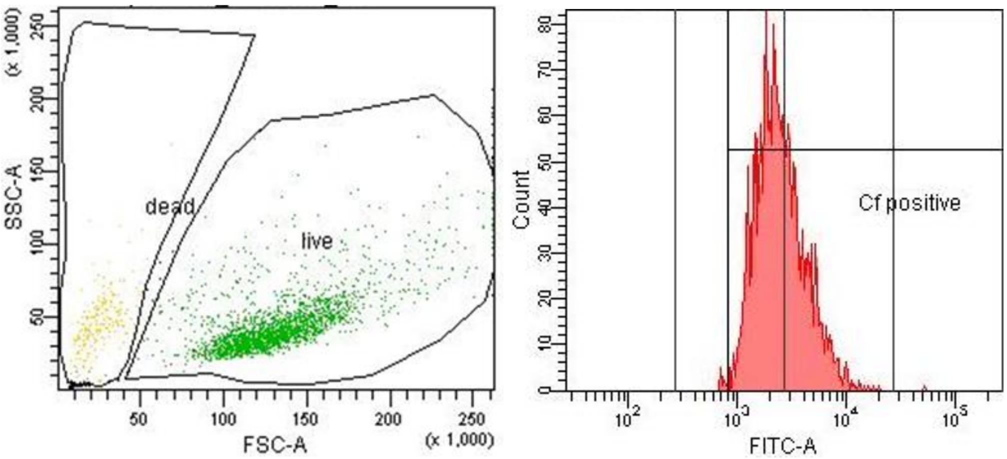

Dabcyl-RRRRWK(Cf)

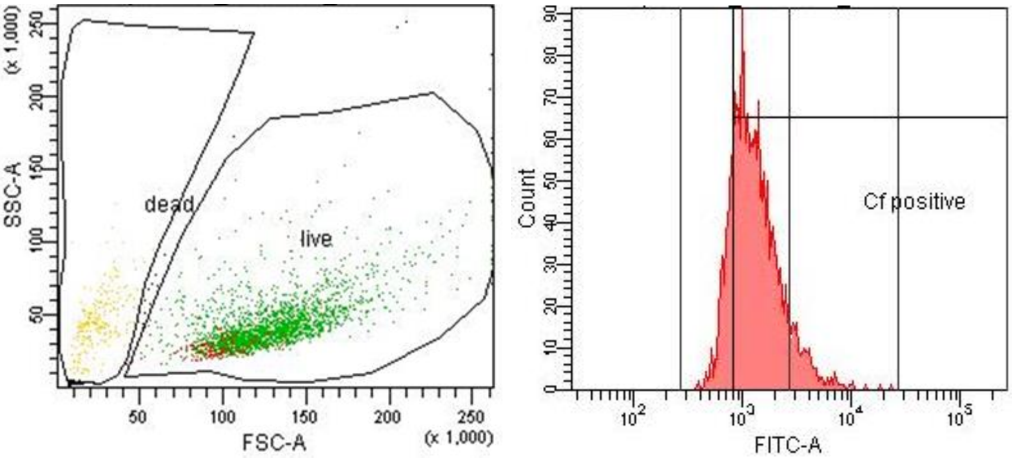

Dabcyl-WRRWRRK(Cf)

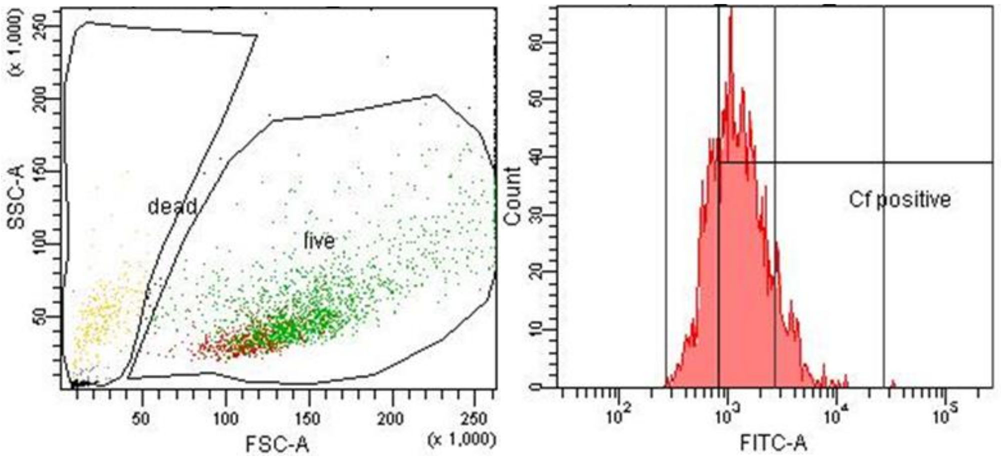

Dabcyl-RR-azaGly-azaGly-RRK(Cf)

Figure S30 Representative histograms and dot plots of cellular uptake measurement. $6.5\cdot10^4$ A-431 cells were treated with peptide solution at 5 μM concentration for 90 min.

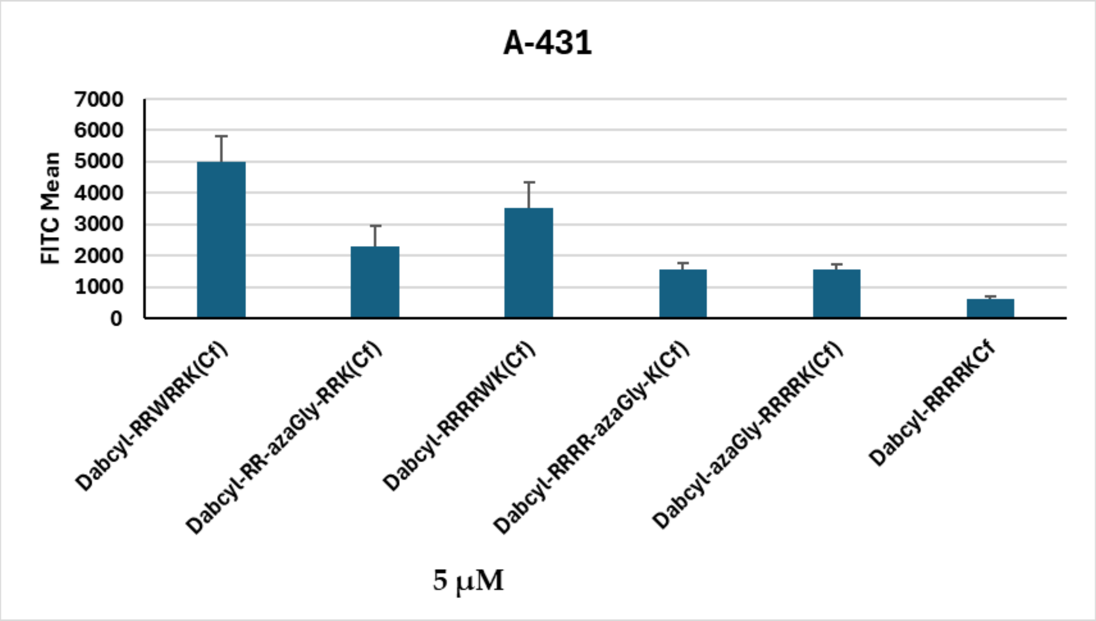

Figure S31 Study of the effect of azaGly substitution of Trp in different positions into Dabcyl-containing tetraarginine derivatives on the cellular uptake. A-431 cells were incubated with 5 μM peptide solution for 90 min at 37 °C. The fluorescence intensity of the cells was determined by flow cytometry. Data represent the mean ± standard deviation (SD).

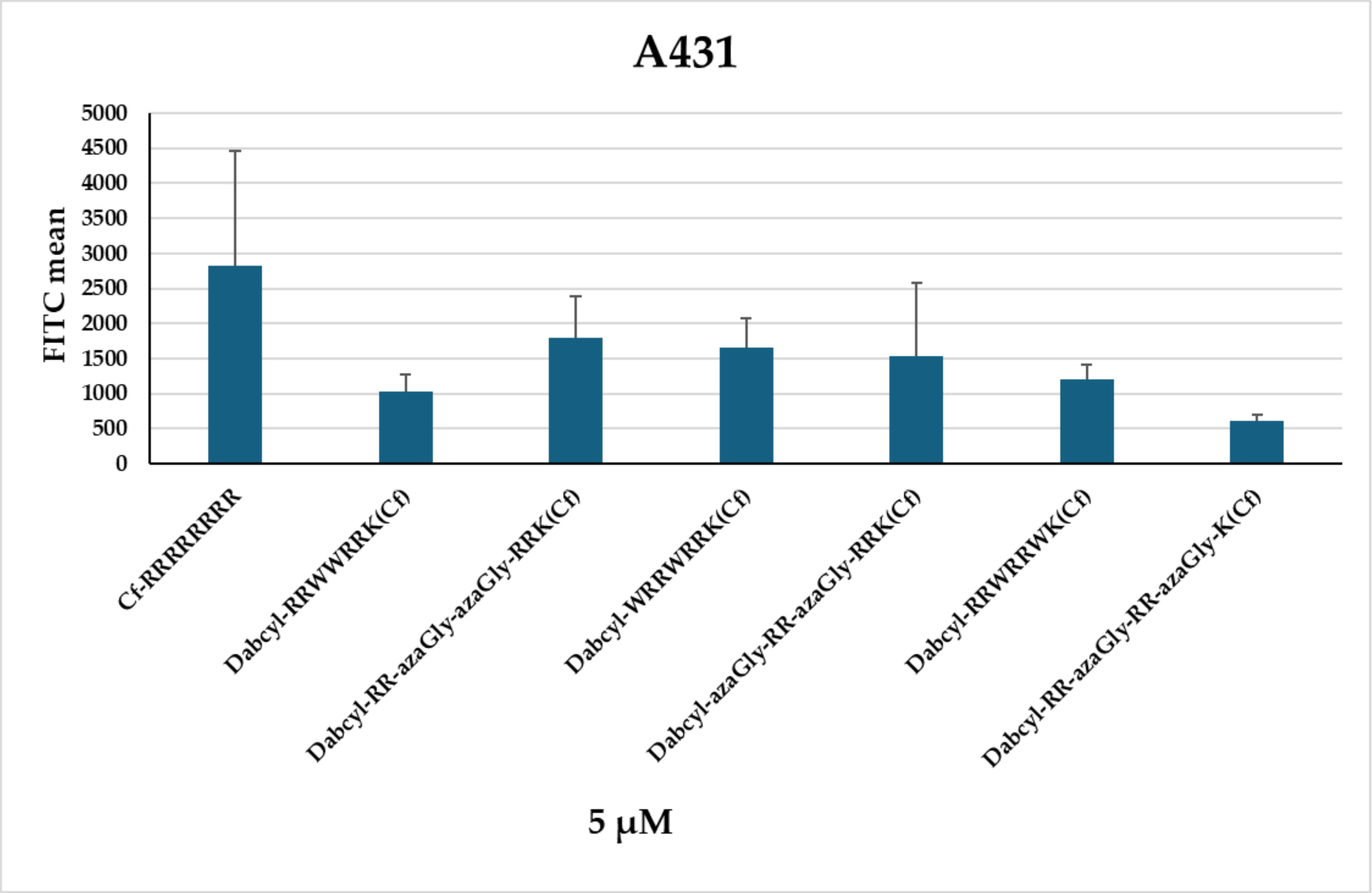

Figure S32 Study of the effect of two azaGly substitution in different positions into Dabcyl-containing tetraarginine derivatives on the cellular uptake. A-431 cells were incubated with 5 μM peptide solution for 90 min at 37 °C. The fluorescence intensity of the cells was determined by flow cy-tometry. Data represent the mean ± standard deviation (SD).
